# Identification and analysis of genes associated with the synthesis of bioactive constituents in *Dendrobium officinale* using RNA-Seq

**DOI:** 10.1038/s41598-017-00292-8

**Published:** 2017-03-15

**Authors:** Chenjia Shen, Hong Guo, Hailing Chen, Yujun Shi, Yijun Meng, Jiangjie Lu, Shangguo Feng, Huizhong Wang

**Affiliations:** 1 College of Life and Environmental Science, Hangzhou Normal University, Hangzhou, 310036 China; 2 Zhejiang Provincial Key Laboratory for Genetic Improvement and Quality Control of Medicinal Plants, Hangzhou Normal University, Hangzhou, 310036 China; 3 Department of Geratology, Hangzhou Hospital of Traditional Chinese Medicine, Hangzhou, 310007 China; 4 School of Foreign Languages, Zhejiang Gongshang University, Hangzhou, 310018 China

## Abstract

*Dendrobium officinale* L. is an important traditional herb with high commercial value in China. Several bioactive constituents, including polysaccharides and alkaloids, reportedly make major contributions toward the excellent medicinal effect of *D. officinale*. In this study, the contents of polysaccharides and alkaloids in various organs of *D. officinale* were measured and compared. We took advantage of transcriptomes from four organs to explore biological mechanisms in the organ-specific distribution of active ingredients in *D. officinale*. Based on Kyoto Encyclopedia of Genes and Genomes pathways, unigenes related to the enzymes involved in fructose and mannose metabolism and unigenes associated with putative upstream elements of the alkaloid biosynthetic pathway were identified. A large number of candidates, including 35 full-length glycosyltransferase genes and 49 full-length P450 genes, were also identified based on the transcriptome data, and the organ-specific expression pattern of these genes was determined. Furthermore, differential expression of all candidate genes was analyzed in two *Dendrobium* species, *D. nobile* L. and *D. officinale*. The data will supply important clues to exploit useful genes involved in polysaccharide and alkaloid synthesis.

## Introduction

The *Dendrobium* genus of plants, the second largest genus in the Orchidaceae, are medicinal herbs with high commercial value in China and other Asian countries^1, 2^. *Dendrobium officinale* L., an endangered and rare orchid in the wild, has been used as folk medicine for immune regulatory for hundreds of years in China. Several active ingredients of *D. officinale*, such as polysaccharides, flavones and alkaloids, have important medicinal effects^3, 4^.

Previous studies focused on characterization of polysaccharides and alkaloid components in *Dendrobium* ^3^. In recent years, soluble polysaccharides were isolated and extracted from the stems of various *Dendrobium* species^5, 6^. In *D. officinale*, most water-soluble polysaccharides, comprising glucose, mannose, arabinose and xylose, are contained in the stems^7^. Soluble polysaccharides show strong bioactivity and are a major market indicator of *D. officinale* quality^8^. Fructose and mannose are the fundamental building blocks for polysaccharide synthesis in *D. officinale* ^3^. Using these monosaccharide units, a series of glycosyltransferases (GTs) catalyze the transfer of sugar moieties from activated donor molecules to specific acceptor molecules to form glycosidic bonds^9^.

Some components and contents of alkaloids in *D. officinale* have been clarified^10^. In *D. officinale*, most alkaloids belong to the terpenoid indole alkaloid (TIA) category^11^. A conserved upstream biosynthetic pathway of TIA exists in various plant species and generates a strictosidine backbone for downstream products^12, 13^. The biosynthetic pathway of TIAs consists of the shikimate, mevalonate (MVA) and methylerythritol phosphate (MEP) pathways, and a series of key enzymes involved in the TIA biosynthetic pathway have been identified^14, 15^. Following strictosidine, the biosynthesis of alkaloids in *D. officinale* may be catalyzed by a set of monooxygenases and hydroxylases^11^. The cytochrome P450s, which play important roles in monooxygenation and hydroxylation reactions, may be involved in the synthesis of alkaloids^16^. The family members and expression patterns of P450 genes in *D. officinale* remain unknown.

Most of the known medicinal ingredients from *D. officinale* are the intermediate or final products of secondary metabolite biosynthesis^17^. Although the bioactivities, molecular structures and composition of polysaccharides and alkaloids from *Dendrobium* have been partially elucidated, the key enzyme-encoding genes involved in their synthesis and metabolic pathways are largely unknown. Thus far, several transcriptomes of *D. officinale* have been sequenced and published^1, 3, 11^; however, there is limited information on the genes involved in the synthesis of major active ingredients and their organ-specific expression patterns. In our study, we established four transcription databases from the leaves, roots, stems and flowers of *D. officinale* and identified a large number of genes involved in polysaccharide and alkaloid biosynthesis. Metabolites exist in various *Dendrobium* species, although their contents may vary largely between different species, the basic biosynthetic pathways are considered to be universal in the genus. Our data should be a useful resource to enhance the biosynthesis of polysaccharides and alkaloids in other spp. of *Dendrobium*.

## Materials and Methods

### Experiment design, plant materials and total RNA extraction

**Figure 1:**
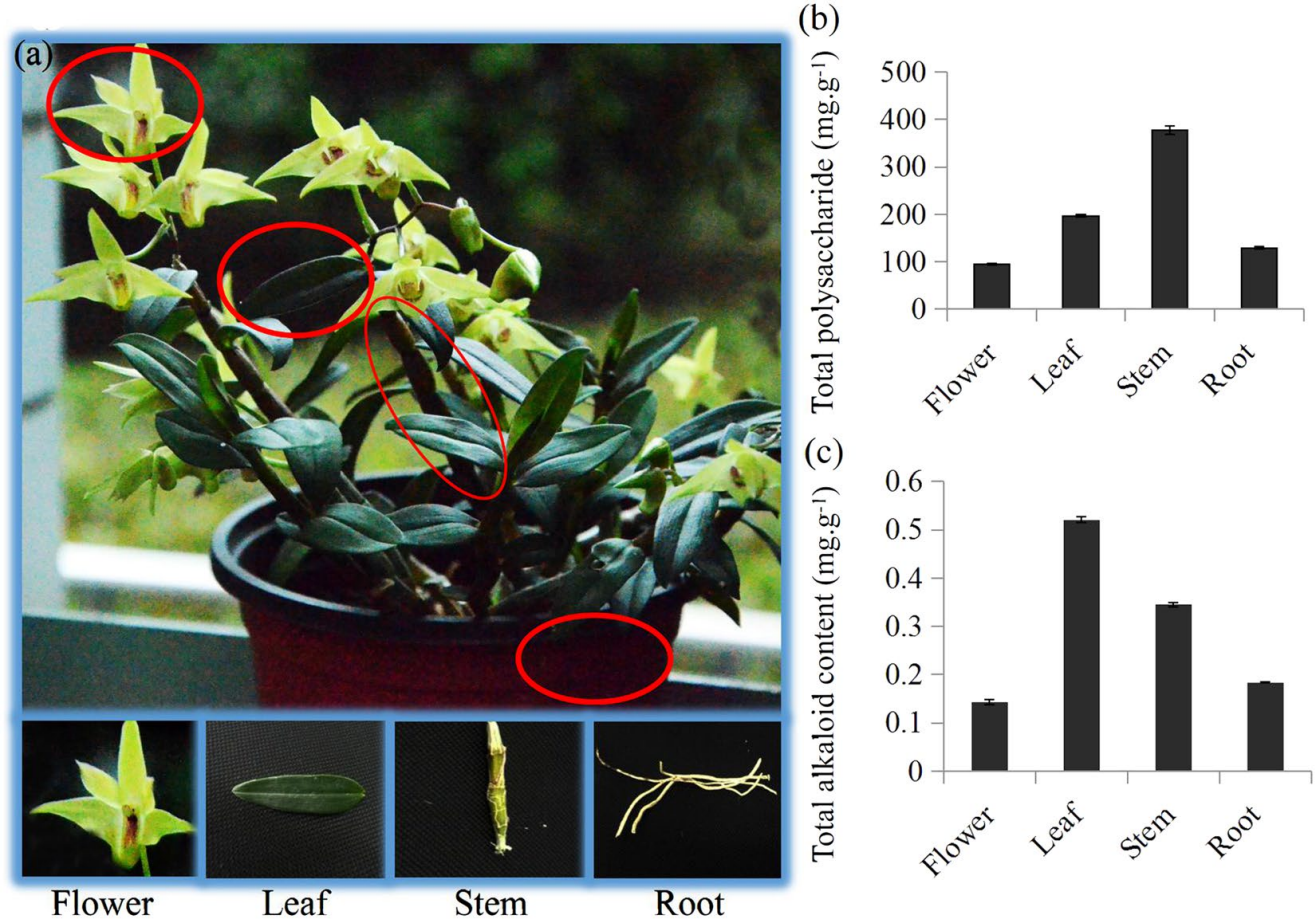
Determination of polysaccharide and alkaloid contents in four different organs of *D. officinale* L. (**a**) Samples from different organs of *D. officinale* L. (**b**) Determination of polysaccharide contents in four organs, including flower, leaf, stem and root, of *D. officinale* L. (**c**) Determination of alkaloid contents in four organs, including flower, leaf, stem and root, of *D. officinale* L.

*Dendrobium officinale* plants were grown in a greenhouse of Hangzhou Normal University, Hangzhou, China. The plants were transferred into independent pots and kept in the greenhouse at a temperature of 25 ± 1 °C with a light/dark cycle of 12/12 h and 60–70% relative humidity. Leaves, stems and roots were collected from 6-month-old *D. officinale* plants and flowers were collected from plants at the flowering stage for paired-end transcriptome sequencing. There were two biological replicates for each organ (Fig. 1a). Total RNAs were extracted using Trizol reagent (Invitrogen, CA, USA) according to the manufacturer’s protocol, and then quantified using Bioanalyzer 2100 and RNA 6000 Nano LabChip Kit (Agilent, CA, USA) with RIN number >7.0.

### Construction of cDNA libraries for digital gene expression sequencing

For RNA-Seq, a total amount of 10 μg of RNA per sample was used as material for cDNA library preparations. RNAs were subjected to enrichment of poly(A)-tailed mRNAs with poly(T) oligo-attached magnetic beads (Thermo Fisher Scientific, MA, USA) and then fragmented into small pieces using divalent cations at a high temperature. The fragmented RNAs were reverse-transcribed to produce the final cDNA library using mRNA-Seq sample preparation kit (Illumina, San Diego, CA, USA) following the manufacturer’s procedure. To preferentially select cDNA fragments of approximately 200–400 bp, all fragments were purified with the AMPure XP system (Beckman Coulter, Beverly, USA). Then, USER Enzyme (NEB, Ipswich, MA, USA) was used with size-selected and adaptor-ligated cDNA at 37 °C for 15 min followed by 5 min of 95 °C treatment. Finally, PCR was performed with High-Fidelity DNA polymerase and PCR products were purified using AMPure XP system. An Agilent Bioanalyzer 2100 system was used for library quality control.

### Sequencing, quality control and *de novo* assembly

The paired-end sequencing was performed on the Illumina Hiseq2500 platform following the recommended protocol and 100/50-bp paired/single-end reads were generated. Raw reads of fastq format were processed by in-house perl scripts. The reads containing adapter or poly-N and reads of low quality were removed from raw data to generate clean reads for the further analyses. Based on the clean reads, the Q20, Q30, GC-content and sequence duplication level of the clean data were calculated. A *de novo* strategy was employed to assemble the transcriptome of *D. officinale* based on a total of 34.3 Gb of data using the Trinity assembly program with default parameters to form contigs.

### Sequence annotation

To identify transcripts homologous to model species, the functions of unigenes were annotated by a locally installed BLASTall program with an *E*-value threshold of 10^−10^ to protein databases, including NCBI non-redundant (nr) (http://www.ncbi.nlm.nih.gov/protein/), Swiss-Prot protein (http://www.uniprot.org/) and Kyoto Encyclopedia of Genes and Genomes (KEGG) databases. Proteins with highest sequence similarity were retrieved for next step annotation. Annotation of the metabolic pathway was produced by KEGG, and Gene Ontology (GO) classifications were carried out by Blast2GO. In detail, sequence alignment via Blastx with the Nr database with default parameters: *e*-value threshold of 1*e* ^−3^ and a recovery of 20 hits per sequence (hsp). For GO and KEGG annotation, the hsp filter was set to 33 to avoid hits where the length of the matching region is smaller than 100 nucleotides. Then, the value of annotation cutoff parameter is set to 60 in our study.

### Expression level calculation of the transcripts and differentially expressed gene (DEG) analysis

An alignment software, Bowtie (version 0.12.7), was applied to map clean reads to all the assembled transcripts by the ‘single-end’ method with parameter ‘-v 3 -a –phred64-quals’. The number of mapped clean reads for each unigene was then counted and normalized into a reads per kb per million reads (RPKM) to calculate the expression level of the unigene. Two data sets from the same organ were treated as a group, and differential expression analysis of two groups was performed using the DESeq R package (version 1.10.1). In this study, the false discovery rate (FDR) was used to calculate the threshold *P*-value in significance tests, and then the results of *P*-values were adjusted by Benjamini and Hochberg’s method. We used an FDR < 0.001 and *P* < 0.05 as the threshold to determine significant differences in comparisons between two samples. A negative binomial distribution-based model was used for DESeq to determine statistical routines for determining differential expression in DEG data. All DEGs were grouped into 25 clusters by a K-means algorithm using MultiExperiment Viewer (MeV) (version 4.9.0) basing on their log_2_ values of transcript abundances.

### Phylogenetic tree building

Multiple sequence alignments were performed on the predicted protein sequences of P450 family using ClustalW with default parameters. Briefly, the gap open penalty parameter was 15, gap extension penalty parameter was 6.66 and the weight matrix selected was ‘IUB’ for DNA. The alignments were visualized subsequently by GeneDoc (http://www.nrbsc.org/gfx/genedoc/), and a phylogenetic tree constructed with aligned 49 dof-P450 protein sequences using MEGA6.1 (http://www.megasoftware.net/) employing the neighbor-joining method.

### Real-time PCR validation

In the experiment of tissue-specific expression confirmation, a *dof-ACTIN* gene was used as an internal standard to calculate relative fold differences basing on the comparative cycle threshold (2^−ΔΔCt^) values. The qRT-PCR procedure was as follows: 1 μL of a 1/10 dilution of cDNA was added to 5 μL of 2 × SYBR^®^ Green buffer, 0.1 μM of each primer and ddH_2_O to a final volume of 10 μL; then, treated with 95 °C for 10 min, 40 cycles of 95 °C for 15 s, and 60 °C for 60 s. All the primer sequences are listed in Table S1. Histogram representation was performed using the average value to visualize tissue-specific expression levels.

### Determination of polysaccharides and total alkaloids in *D. officinale*

Leaf, stem and root samples were collected from 6-month-old *D. officinale* and flower samples from plants at the flowering stage. The phenol-sulfuric acid method was applied to determine the polysaccharide contents in different four samples.

For alkaloids, all samples were soaked five times with 90% ethanol for 12 h, and then heated to boiling for 2 h. The alcohol extract was concentrated with nonalcoholic, and then dissolved with 5% hydrochloric acid. After leaching, aqueous acid solution was extracted three times by petroleum, and the pH value adjusted to 10 using strong aqueous ammonia. The alkaline solution was extracted twice with chloroform, concentrated, and the total alkaloids obtained. The total alkaloids are quantified according to Zhang’s description^18^.

### Statistical analysis

Differences between values were calculated using one-way ANOVA with Student’s *t*-test at *P* < 0.05 in Excel software. All expression analysis was performed for five biological repeats and figures show the average values of five repeats.

## Results

### Determination of polysaccharides and alkaloids in four different organs

To provide a scientific basis for utilization of limited *Dendrobium* resources, contents of total polysaccharides and total alkaloids were determined in various organs of *D. officinale*. Polysaccharides and alkaloids were determined in all four organs including flowers (F), leaves (L), stems (S) and roots (R), with significant differences in contents of the two components between the different organs. Polysaccharides were mainly concentrated in stems, while alkaloids were at high concentrations in leaves (Fig. 1b,c). The highest content of total polysaccharides was 377.6 mg.g^−1^ for stems and the highest content of total alkaloids was 0.52 mg.g^−1^ for leaves. We took advantage of our transcriptomes from the four organs to explore biological mechanisms in organ-specific distribution of active ingredients in *D. officinale*.

### Identification and clustering of organ-specific expression of genes

**Figure 2:**
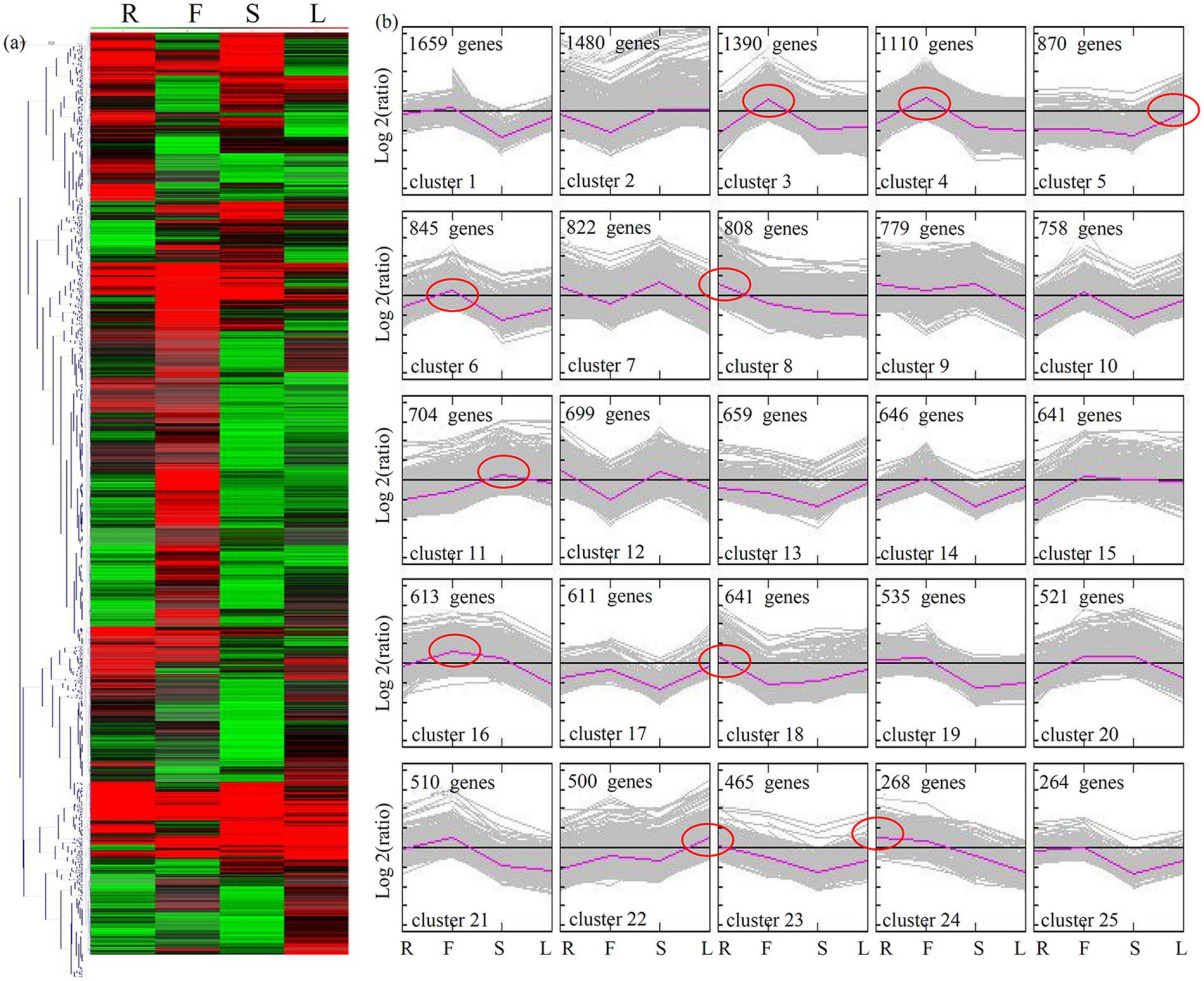
MeV cluster analysis of organ-differentially expressed genes. (**a**) Heat map illustrating the expression profiles of the organ-differentially expressed genes. (**b**) Cluster analysis by the K-means method from the gene expression profiles. Red circles indicated the organ-specific expressed genes.

The raw data were published in our previous study^1^. Based on the RNA-Seq data, the RPKM values were used to calculate the differential expression of each unigene between different organ samples. A total of 18,721 significant DEGs were identified and analyzed using criteria of 10-fold differences and p < 0.05 (Fig. 2a). To reflect the major trends and organ-specific expression between different organs in *D. officinale*, all DEGs were assigned to 25 clusters by K-means method and hierarchical clustering. Transcripts of most DEGs were detectable in different organs in *D. officinale*. Unigenes belonging to clusters 3, 4, 6 and 16 were more highly expressed in flowers than other organs; unigenes belonging to clusters 8 and 18 were highly expressed in flowers; and transcript levels of unigenes in cluster 5 were very high in leaves. Interestingly, all unigenes predominantly expressed in stems belonged to cluster 11 (Fig. 2b).

### GO classification of organ-differentially expressed genes in *D. officinale*

**Figure 3:**
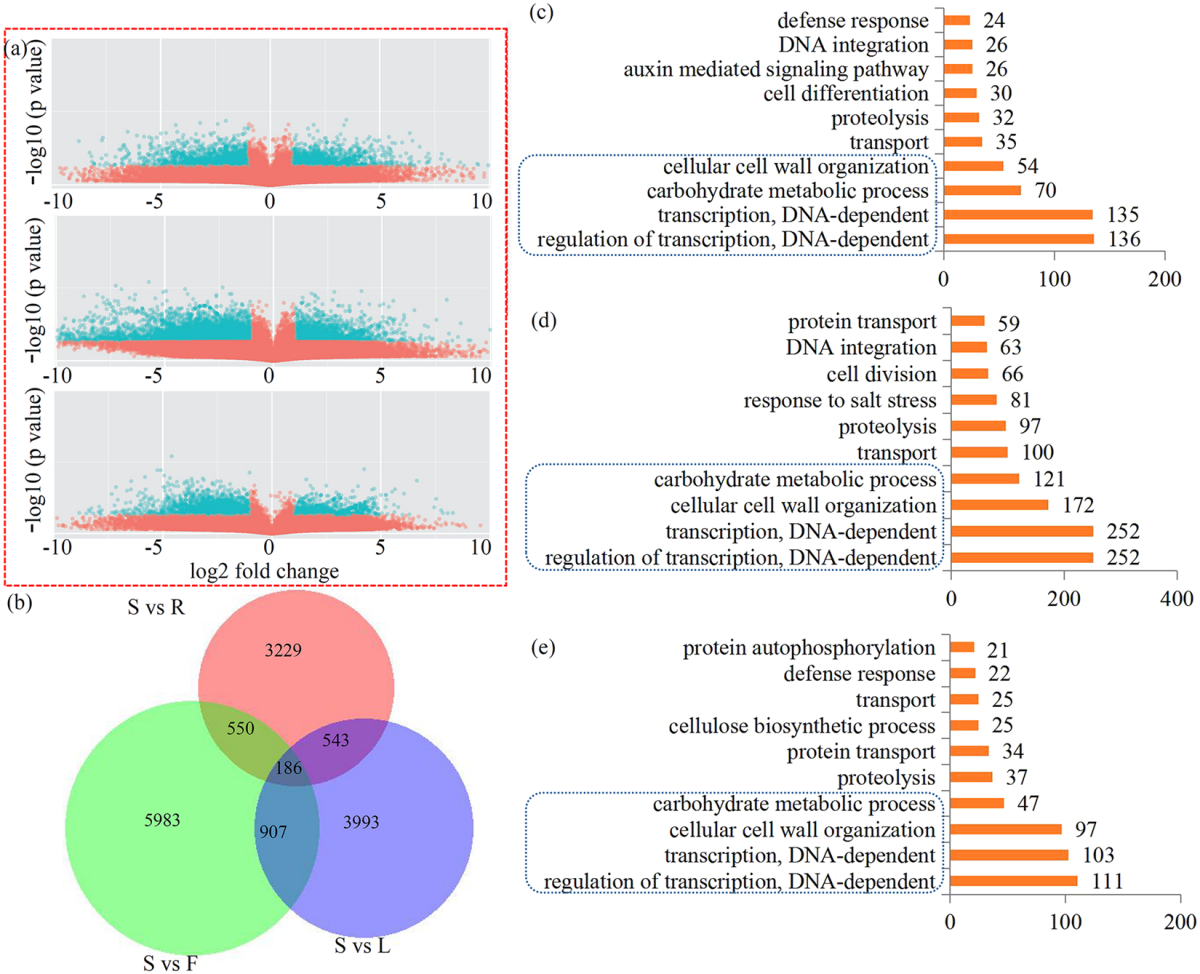
Overview of the differentially expressed unigenes (DEGs) among four different organs in *D. officinale*. (**a**) Volcanoplots of the DEGs in different comparisons, including S vs R, S vs F and S vs L. (**b**) VennDiagrams of the DEGs in different comparisons. Gene Ontology (GO) classification of DEGs in the S vs R comparison (**c**), S vs F comparison (**d**) and S vs L comparison (**e**).

We then identified unigenes that were highly differentially expressed between the stems and any of the other three organs. Based on a criteria of 10-fold differences and p < 0.05, a total of 4508 significant DEGs were identified and analyzed in the ‘S vs. R’ comparison. There were 7625 and 5628 significant DEGs in the ‘S vs. F’ and ‘S vs. L’ comparisons, respectively (Fig. 3a). We compared the three data sets from different comparisons using a Venn diagram. Most of the DEGs in the three comparisons were independent of each other. In detail, 736 DEGs were identified in both ‘S vs. R’ and ‘S vs. F’ comparisons; 1093 DEGs were identified in both ‘S vs. F’ and ‘S vs. L’ comparisons; while 729 DEGs were identified in both ‘S vs. R’ and ‘S vs. L’ comparisons. There were 186 DEGs identified in all three comparisons (Fig. 3b). Furthermore, to demonstrate valuable information concerning the stem-specifically expressed genes in *D. officinale*, we assigned GO terms to the DEGs in different comparisons. The top 10 enriched GO terms in each comparison are shown in Fig. 3c–e. GO term enrichment analysis indicated that the genes were involved in various biological processes and molecular functions. Interestingly, four GO terms, regulation of transcription, transcription, carbohydrate metabolic process and cellular cell wall organization, were significantly enriched in DEGs of all three comparisons.

### Metabolic pathway assignment by KEGG

We mapped a large number of stem-differentially expressed genes to reference canonical pathways in KEGG. A total of 936 significant DEGs in the ‘S vs. R’ comparison were assigned to 163 KEGG pathways (Table S2). Among these KEGGs, 70 of 163 showed significant differences (*P* < 0.01) between stem and root samples. The metabolic pathways represented the greatest group, with most DEGs involved in starch and sucrose metabolism (35 DEGs), cyano-amino acid metabolism (27 DEGs), pyruvate metabolism (24 DEGs), glyoxylate and dicarboxylate metabolism (20 DEGs), purine metabolism (18 DEGs) and glycine, serine and threonine metabolism (17 DEGs). A total of 2274 DEGs in the ‘S vs. F’ comparison were assigned to 174 KEGG pathways (Table S3). Among these KEGGs, 107 of 174 showed significant differences (*P* < 0.01) between stem and flower samples. A large number of DEGs were also identified in several metabolic pathways, such as starch and sucrose metabolism (70 DEGs), pyruvate metabolism (58 DEGs), cyano-amino acid metabolism (47 DEGs) and fructose and mannose metabolism (43 DEGs). Finally, a total of 3534 DEGs in the ‘S vs. L’ comparison were assigned to 177 KEGG pathways, and 167 of these showed significant differences (*P* < 0.01) between stem and leaf samples (Table S4). In total, 113 DEGs were involved in glycolysis, 93 DEGs in starch and sucrose metabolism, 81 in pyruvate metabolism and 74 in purine metabolism.

### Putative pathway for fructose and mannose metabolism in *D. officinale*

**Figure 4:**
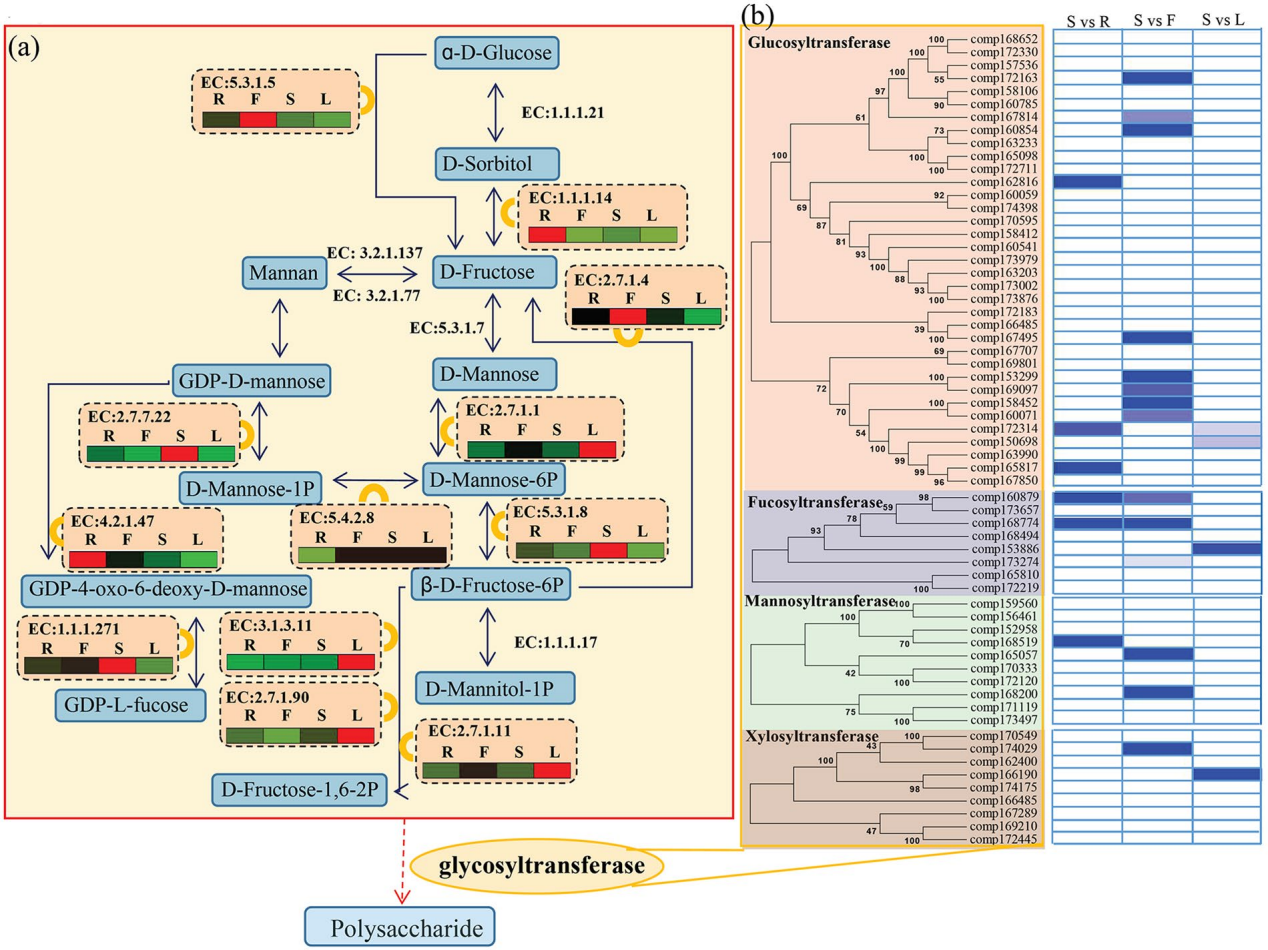
Expression patterns of the putative fructose and mannose metabolism unigenes in *D. officinale*. (**a**) The pathway of fructose and mannose metabolism is based on KEGG analysis. The full names of enzymes by EC IDs are provided in Table S5. The average expression level of the enzyme encoding unigenes in various organs is indicated by a heat map. The grids with different colors from green to red show the relative expression levels to maximum RPKM values, from 0 to 100%, respectively. (**b**) Significance analysis of glycosyltransferase unigenes in three stem contained comparisons, including S vs R, S vs F and S vs L. The grids with different shades of blue show the different significant values, from 0 to 0.01.

Based on KEGG pathways, 54 unigenes encoding 12 key enzymes involved in the fructose and mannose metabolism were identified. A detailed metabolic map with expression patterns of these key genes encoding enzymes for fructose and mannose metabolism was constructed (Fig. 4a). Each enzyme in the pathway was associated with several unigenes (Table S5). The largest number of unigenes (12 unigenes) were identified as fructokinase (EC: 2.7.1.4) encoding genes; and the second largest number of unigenes (eight) were annotated as 6-phosphofructokinase (EC: 2.7.1.90) encoding genes; while the third largest number of unigenes (seven) were annotated as hexokinase (EC: 2.7.1.1) encoding genes. Furthermore, six unigenes were annotated as encoding fructose-1,6-bisphosphatase (EC: 3.1.3.11), four encoding mannose-6-phosphate isomerase (EC: 5.3.1.8), another four encoding L-iditol, 2-dehydrogenase (EC: 1.1.1.14) and three encoding pyrophosphate-fructose-6-phosphate 1-phosphotransferase (EC: 2.7.1.90). In this pathway, xylose isomerase (EC: 5.3.1.5), mannose-1-phosphate guanylyltransferase (EC: 2.7.7.22), phospho-mannomutase (EC: 5.4.2.8), GDP mannose 4,6-dehydratase (EC: 4.2.1.47) and GDP-L-fucose synthase (EC: 1.1.1.271) were each associated with two unigenes respectively.

The average expression levels of the unigenes associated with each enzyme were calculated based on RPKM values. The genes encoding three enzymes, mannose-1-phosphate guanylyltransferase, GDP-L-fucose synthase and mannose-6-phosphate isomerase, were predominantly expressed in stems.

### Identification of GT-encoding genes in *D. officinale*

GTs are a very widespread group of carbohydrate-active related enzymes, and participate in glycan and glycoside biosynthesis in higher plants^19^. A total of 280 glycosyltransferase genes, including 236 glucosyltransferase genes, 11 fucosyltransferase genes, 16 mannosyltransferase genes and 17 xylosyltransferase genes, were identified using BLASTX (e < 0.00001) in four of the transcriptomes (Table S6). Among these sequences, 35 full-length GT genes, eight fucosyltransferase genes, 10 mannosyltransferase genes and nine xylosyltransferase genes were isolated for phylogenetic tree construction. The significant differences in the expression of these genes between stems and the other three organs were also analyzed (Fig. 4b). Six GT genes showed significant differences in expression between stems and roots; 13 GT genes had significant differences in expression between stems and flowers; and only four GT genes had significant differences in expression between stems and leaves.

### Putative alkaloid biosynthetic and metabolic pathway in *D. officinale*

**Figure 5:**
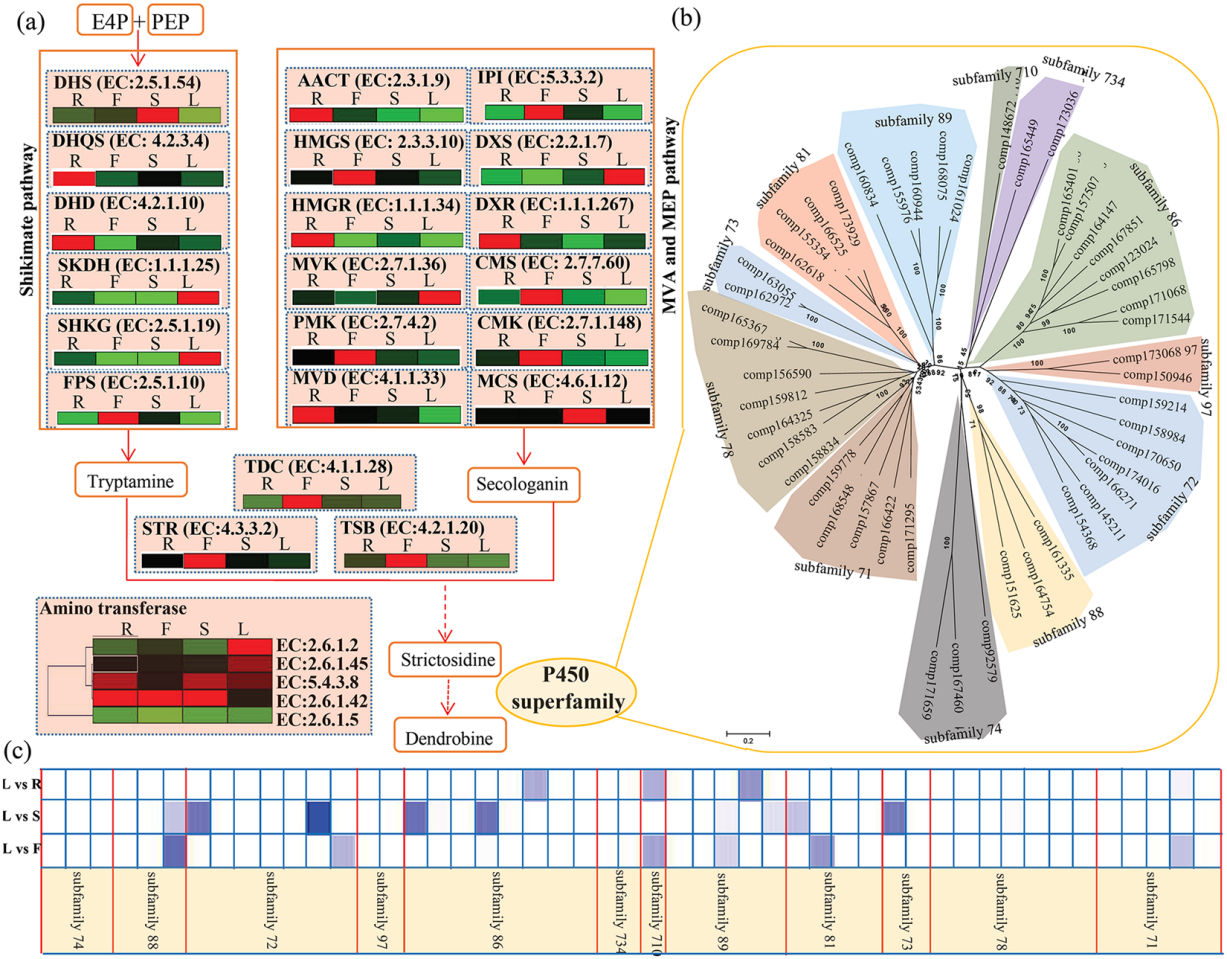
Expression pattern of the unigenes associated with putative alkaloid biosynthesis in *D. officinale*. (**a**) Expression pattern of the unigenes associated with putative upstream elements of alkaloid biosynthetic pathway. Full names of enzymes represented by their abbreviated names were showed in Table S7. The average expression level of the enzyme encoding unigenes in various organs is indicated by a heat map. The grids with different colors from green to red show the relative expression levels to maximum RPKM values, from 0 to 100%, respectively. (**b**) Phylogenetic analysis of all P450 genes with full-length cDNA from *D. officinale*. Branches in different background colors indicated different subfamilies. (**c**) Significance analysis of P450 genes in three leaf contained comparisons, including L vs R, L vs S and L vs F. The grids with different shades of blue show the different significant values, from 0 to 0.01.

The primary type of alkaloid in *D. officinale* extracts was Terpenoid indole alkaloid^11^, and therefore the unigenes annotated as elements of TIA biosynthesis were screened and the expression levels of the unigenes were mapped to the pathway. In our study, unigenes involved in the putative upstream elements of the alkaloid biosynthetic pathway, which originates from the shikimate, MVA and MEP pathways, were identified in *D. officinale*. Transcripts of the enzyme-encoding genes involved in the shikimate, MVA and MEP pathways are shown in Fig. 5a. In total, 17 unigenes associated with six enzymes were targeted to the shikimate pathway, including 3-deoxy-D-arabinoheptulosonate-7-phosphate synthase (DHS, EC: 2.5.1.54), 3-dehydroquinate synthase (DHQS, EC: 4.2.3.4), 3-dehydroquinate acid dehydratase (DHD, EC: 4.2.1.10), shikimate dehydrogenase (SKDH, EC: 1.1.1.25), 5-enolpyruvylshikimate-3-phosphate synthase (SHKG. EC: 2.5.1.19) and famesyl diphosphate synthase (FPS, EC: 2.5.1.10). Moreover, 36 unigenes associated with 12 enzymes were located to MVA and MEP pathways. Detailed information on these enzyme-encoding genes is listed in Table S7. Importantly, a series of key enzyme-encoding genes involved in strictosidine were identified. There were unigenes annotated as encoding β-subunit of tryptophan synthase (TBS, EC: 4.2.1.20); five unigenes as encoding tryptophan decarboxylase (TDC, EC: 4.1.1.28); and nine unigenes as encoding strictosidine synthase (STR, EC: 4.3.3.2) (Table S7). There were 43 putative transcripts annotated as associated with five independent transaminases (Table S8).

The average expression level of the unigenes associated with each enzyme was also determined, based on RPKM values. The genes encoding six enzymes, including SHDH, SHKG, MVK (EC: 2.7.1.36), DXS (EC: 2.2.1.7) and amino transferases (EC: 2.6.1.2 and EC: 2.6.1.45), were predominantly expressed in leaves.

### Identification of P450 superfamily genes in *D. officinale*

In our study, 236 sequences were identified as putative P450 superfamily members (Table S9). Among these sequences, 49 were isolated as full-length cDNA of P450 genes. To investigate the relationship and classification of P450 family genes in *D. officinale*, a phylogenetic tree was constructed for all P450 genes with full-length cDNA. The results indicated that these genes could be grouped into 12 major subfamilies based on the classical nomenclature of the P450 superfamily^20^. In detail, the largest subfamily (subfamily 86) consisted of eight P450 genes and the second largest subfamilies (subfamilies 72 and 78) consisted of seven P450 genes (Fig. 5b).

The significant differences in the expression of full-length P450 genes between leaves and the other three organs are shown in Fig. 5c. Six full-length P450 genes showed significant differences in expression between leaves and flowers; seven full-length P450 genes showed significant differences in expression between leaves and stems; and only three full-length P450 genes had significant differences in expression between leaves and roots.

### Content determination of polysaccharides and expression analysis of key polysaccharide genes in two *Dendrobium* species

**Figure 6:**
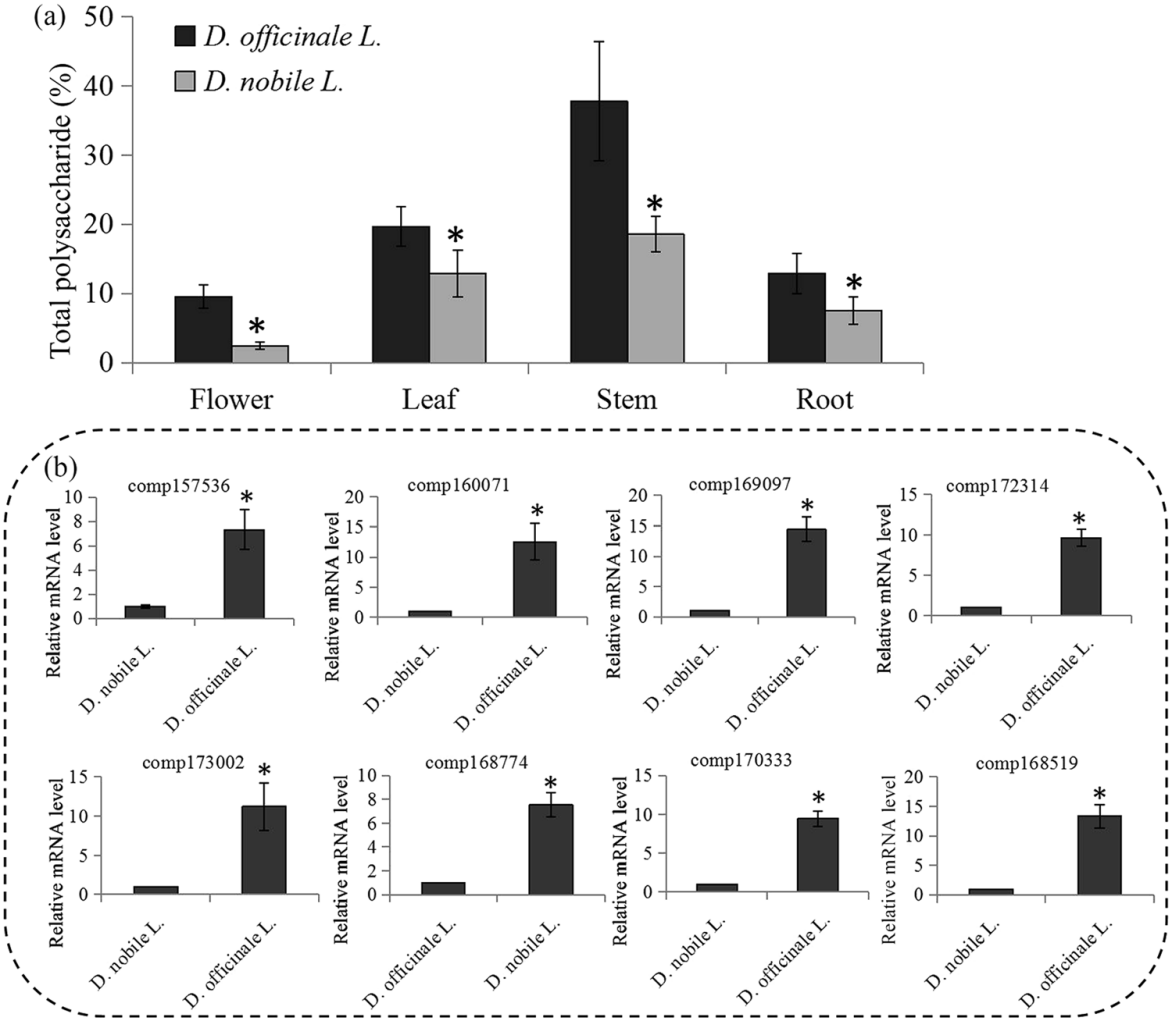
Polysaccharide contents determination and expression analysis of key polysaccharide biosynthetic genes in two different *dendrobium* species. (**a**) The polysaccharide contents were measured in four organs of two different dendrobium species (*D. nobile* L. and *D. officinale* L.). The significantly changes in polysaccharide contents between *D. nobile* L. and *D. officinale* L. were indicated by “*”. (**b**) The related expression levels of eight *D. officinale* L. highly expressed genes. The significantly changes in expression levels of polysaccharide genes between *D. nobile* L. and *D. officinale* L. were indicated by “*”.

We measured the total polysaccharides in four organs of two *Dendrobium* species: *D. nobile* and *D. officinale*. The polysaccharide level was higher in *D. officinale* than in *D. nobile* (Fig. 6a).

To validate the key enzyme-encoding genes possibly involved in polysaccharide synthesis, two groups of cDNA libraries were respectively prepared from the two *Dendrobium* species. The cDNA libraries from the stem samples of the two species were used to analyze the expression of 62 GT-encoding genes by qRT-PCR method. In total, eight GT-encoding genes were mainly expressed in *D. officinale* (the species with higher polysaccharide content) (Fig. 6b). Interestingly, among these genes, three GT genes (comp169097, comp160071 and comp172314), one fucosyltransferase gene (comp168774) and one mannosyltransferase gene (comp168159) showed stem-specific expression (Fig. 4).

### Content determination of alkaloids and expression analysis of key alkaloid genes in two *Dendrobium* species

**Figure 7:**
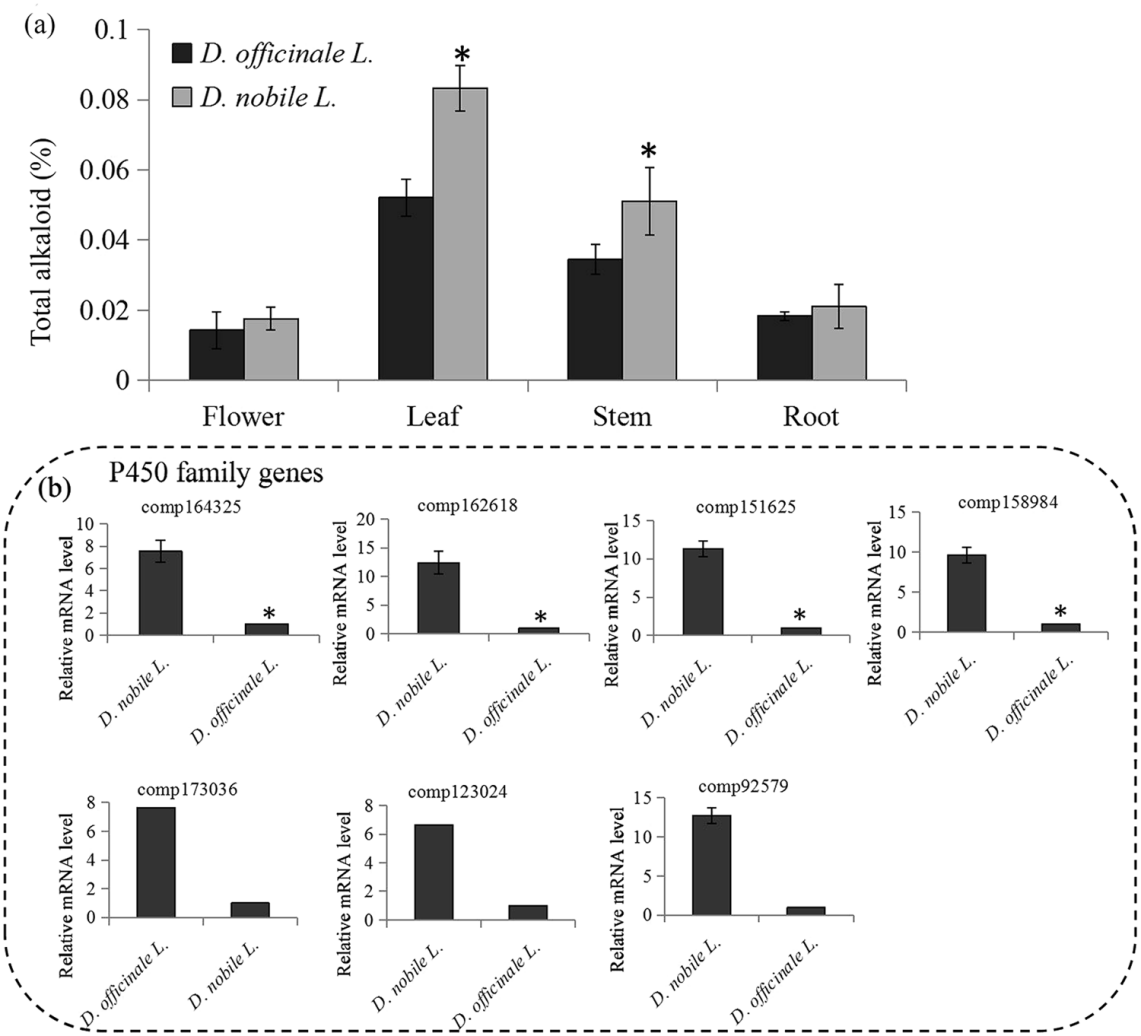
Alkaloid contents determination and expression analysis of key alkaloid biosynthetic genes in two different *dendrobium* species. (**a**) The alkaloid contents were measured in four organs of two different dendrobium species (*D. nobile* L. and *D. officinale* L.). The significantly changes in polysaccharide contents between *D. nobile* L. and *D. officinale* L. were indicated by “*”. (**b**) The related expression levels of seven *D. nobile* L. highly expressed genes. The significantly changes in expression levels of polysaccharide genes between *D. nobile* L. and *D. officinale* L. were indicated by “*”.

We measured the total alkaloids in four organs of *D. nobile* and *D. officinale*. The alkaloid level was lower in the leaves and stems of *D. officinale* than of *D. nobile*. There were no significant differences in flowers and roots between the two species (Fig. 7a).

To validate the key enzyme-encoding genes possibly involved in alkaloid synthesis, two groups of cDNA libraries were also prepared from the two species, respectively. The cDNA libraries from the leaf samples of the two species were used to analyze the expression of 49 P450 genes. Seven P450 genes were expressed more in *D. nobile* than in *D. officinale* (over five-fold): comp164325, comp162618, comp151625, comp158984, comp173036, comp123024 and comp92579 (Fig. 7b). Among these genes, three had leaf-specific expression: comp151625, comp158984 and comp123024 (Fig. 5c).

## Discussion

Part of the Orchidaceae family, *D. officinale* is a very popular Chinese herb spread widely in China. Polysaccharides and alkaloids are two of the most important active constituents of *D. officinale*, and a large number of putative polysaccharides and alkaloid biosynthetic genes have been previously identified^3, 11^. However, little is known about the mechanisms responsible for organ-specific distribution and metabolism of these two active constituents. The aim of our study was to identify more genes related to polysaccharide and alkaloid synthesis. To increase the medicinal value of *D. officinale*, our data provided several candidate genes for genetic improvement.

In recent years, the availability of transcriptome data for *D. officinale* has increased. For example, 36,407 unique sequences were generated and assembled by Guo’s group and two RNA-Seq data sets with a total of 145,791 unigenes were published by Zhang’s group^3, 11^. In our study, eight RNA-Seq data sets which could be assigned to 299,107 unigenes were assembled^1^. The number of unigenes obtained from our experiment increased eight-fold compared to that of Guo and two-fold compared to Zhang, indicating more comprehensive information for functional studies of this species.

The stem of *D. officinale* is a major organ containing polysaccharides, which are promising bioactive constituents for use as drugs^6^. Analysis of the stem-specific expressed genes gives us a solid base to understand the accumulation of polysaccharides in stems. In *Arabidopsis*, mannose-1-phosphate guanylyltransferase is required by cellulose biosynthesis and identified as a member of the cellulose synthase family^21^. Several previous studies have reported that cellulose synthase family genes are associated with the biosynthesis of mannan polysaccharides^6, 22^. In *D. officinale*, stem-specific expression pattern of mannose-1-phosphate guanylyltransferase genes suggested their involvement in the biosynthesis of mannan polysaccharides in stem. Furthermore, mannose-6-phosphate isomerase is another key hexose metabolism enzyme that catalyzes reversible isomerization between D-fructose 6-phosphate and D-mannose 6-phosphate^23^. GDP-L-fucose synthase, an enzyme existing widely in animal and plant kingdoms, converts GDP-4-oxo-6-deoxy-D-mannose into GDP-L-fucose^24^. The stem-specific expression pattern of mannose-6-phosphate isomerase and GDP-L-fucose synthase genes suggested their essential roles in the accumulation of fructose, which are the raw materials of polysaccharide biosynthesis, in stems.

The major role for GTs in plant is biosynthesis of glycan and glycoside in cell walls. Transfer of sugar moieties from activated donors to specific acceptors to form glycosidic bonds is a key downstream step for polysaccharide biosynthesis^19^. A large number of GTs were identified in the model plant *Arabidopsis* (more than 450 members) and rice (over 600 members)^25–27^. Polysaccharides from *Dendrobium* are mainly composed of glucose and mannose, as well as tiny amounts of rhamnose, xylose and arabinose^7^. In *D. officinale*, a large number of unigenes were annotated as GT encoding genes and mannosyltransferases encoding genes, providing several candidates that regulate polysaccharide synthesis and secondary metabolism in *D. officinale*. Interestingly, 12 GTs encoding genes and three mannosyltransferases encoding genes significantly expressed in the stem (Fig. 4b). There genes may play an important role in the downstream biosynthesis of polysaccharides.

In *D. officinale*, the highest content of total alkaloids was in leaves (Fig. 1c). The expression patterns of the unigenes annotated as elements of TIA biosynthesis were analyzed using the transcriptome data. The productions of the shikimate pathway in plants are not only essential components of protein synthesis, but are also precursors for a wide range of secondary metabolites^28, 29^. SHKG is a shikimate pathway key enzyme involved in the formation of enolpyruvylshikimate 3-phosphate^30^. In *D. officinale*, the leave-specific expressed SHKG encoding genes may increase the metabolic rate of shikimate pathway to produce more tryptamines, which are precursors for strictosidine biosynthesis. Biosynthesis of secologanin, an important precursor for TIA metabolism, is catalyzed by a series of enzymes associated with the MVA and MEP pathways^11, 31^. In *D. officinale*, the genes encoding MVK and DXS were mainly expressed in leaves, suggesting a role of MVK and DXS in leaf-specific accumulation of alkaloids in *D. officinale*.

Following EST annotation of the 454 EST pool, several cytochrome P450 transcripts were identified by Guo’s group^11^. Some P450 subfamilies belonging to the CYP71 clan were reported to be involved in bioactive secondary metabolism in plants^32^. A few members of P450 subfamily 71 participate in modification of shikimate products and intermediates; some members of P450 subfamily 72 are involved in catabolism of isoprenoid hormones^33^; and some members of subfamily P450 subfamily 86 catalyze hydroxylations of fatty acids^34^. In our study, one gene (comp166422) in subfamily 71, three (comp159214, comp158984 and comp154368) in subfamily 72 and three (comp164147, comp123024 and comp171544) in subfamily 86, showed differential expression between leaves and other organs (Fig. 5b,c). These P450 genes are candidate genes for further identification.

There were large differences in polysaccharide and alkaloid contents between *D. nobile* and *D. officinale* (Figs 6a and 7a). This provided an additional opportunity to validate and screen functional genes involved in the biosynthesis of polysaccharides and alkaloids. These data will supply important clues to exploit useful genes involved in polysaccharide and alkaloid synthesis.

## Electronic supplementary material

Supplementary Information
