# Supplementary material for: Identification and analysis of genes associated with the synthesis of bioactive constituents in Dendrobium officinale using RNA-Seq
Source: Sci Rep. 2017 Mar 15;7:187. doi: 10.1038/s41598-017-00292-8 (PMC5412657; doi:10.1038/s41598-017-00292-8)
Supplement: Supplementary file 1 — Supplementary Information [file 41598_2017_292_MOESM1_ESM.pdf]

Identification and analysis of genes associated with the synthesis of bioactive constituents in *Dendrobium officinale* using RNA-Seq

Chenjia Shen[1,2], Hong Guo[1,2], Hailing Chen[3], Yujun Shi[4], Yijun Meng[1,2], Jiangjie Lu[1,2], Shangguo Feng[1,2*], Huizhong Wang[1,2*]

1. College of Life and Environmental Science, Hangzhou Normal University, Hangzhou 310036, China;

2. Zhejiang Provincial Key Laboratory for Genetic Improvement and Quality Control of Medicinal Plants, Hangzhou Normal University, Hangzhou 310036, China;

3. Department of Geratology, Hangzhou Hospital of Traditional Chinese Medicine, Hangzhou 310007, China;

4. School of Foreign Languages, Zhejiang Gongshang University, Hangzhou 310018, China;

Shangguo Feng

Email: shangguo007@126.com

Huizhong Wang:

Email: whz62@163.com

Table S1 The primer sequences of key genes involved in the polysaccharides and alkaloid synthesis.

gene	UP	DN
comp157536	GCCTCCCCTTCTCCGACGA	CTTTCTTCAGTCCCGGGAT
comp160071	GAGGATGTTGGCATCACCC	GATAACATTCACAAAATA
comp169097	CCAGCTGCTCAAATAAGC	AATTTCTTGTATGCAAATA
comp172314	CAAATAAGAGTGAATATA	CACCAAATCCTTTACAAC
comp173002	CAAACGAAGACCCTCCTAC	CGACATGGCCGAGATTTG
comp168774	CAGCAAATTGAATGTAGT	GTCAAGTGTATGGTGGGC
comp170333	CAAATTCTTCTGCTTTTA	GTAATGCACGTTCAGGTCAG
comp168519	GATGATCAGCTGTTGATCG	CGAGCAAGTGATGTCCTTC
comp164325	CTCTCAATTTCTCCGTACC	GCACTTATTCCATCACTCA
comp162618	CTCCAGGATTCTACAGAA	GCGGCGAGTCTCATCCGC
comp151625	GACGTTGGCCGTCGCTGT	CAGCAGCTTTAGGCCATC
comp158984	GAGATATATTGGTGGAAGC	GCCTTGGCTGGCGACCGTA
comp173036	GAAATCCAAAAACAAGCA	GCTTGATAGTTATGAGAT
comp123024	CATAGCAAGTTCTCGACAT	AATTCAAACCGGAGCGGT
comp92579	GTCATTTAACTGATTACA	CTGGGCTGAACGCAACAT

Table S2 The information of 936 significantly DEGs in the 'S vs R' comparison were assigned to 163 KEGG pathways

ko_id	ko_name	pvalue	FDR
ko00460	Cyanoamino acid metabolism	0	0
ko00500	Starch and sucrose metabolism	1.11E-16	9.05E-15
ko04110	Cell cycle	1.19E-14	6.45E-13
ko00562	Inositol phosphate metabolism	1.26E-12	4.10E-11
ko00630	Glyoxylate and dicarboxylate metabolism	1.02E-12	4.10E-11
ko04070	Phosphatidylinositol signaling system	1.40E-11	3.80E-10
ko04115	p53 signaling pathway	3.10E-11	6.31E-10
ko00260	Glycine, serine and threonine metabolism	2.89E-11	6.31E-10
ko00905	Brassinosteroid biosynthesis	1.76E-10	3.19E-09
ko04111	Cell cycle - yeast	2.51E-10	4.08E-09
ko00620	Pyruvate metabolism	1.37E-09	2.02E-08
ko00010	Glycolysis / Gluconeogenesis	2.38E-09	3.23E-08
ko04666	Fc gamma R-mediated phagocytosis	3.15E-09	3.94E-08
ko00710	Carbon fixation	8.32E-09	8.47E-08
ko04144	Endocytosis	7.31E-09	8.47E-08
ko03420	Nucleotide excision repair	8.32E-09	8.47E-08
ko00270	Cysteine and methionine metabolism	1.09E-08	1.05E-07
ko00051	Fructose and mannose metabolism	1.58E-08	1.43E-07
ko03030	DNA replication	2.26E-08	1.94E-07
ko04114	Oocyte meiosis	5.09E-08	4.15E-07
ko04120	Ubiquitin mediated proteolysis	6.20E-08	4.81E-07
ko00360	Phenylalanine metabolism	2.94E-07	2.18E-06
ko04020	Calcium signaling pathway	1.43E-06	1.01E-05
ko00670	One carbon pool by folate	3.22E-06	2.19E-05
ko00280	Valine, leucine and isoleucine degradation	3.94E-06	2.57E-05
ko05213	Endometrial cancer	6.71E-06	4.21E-05
ko04510	Focal adhesion	7.61E-06	4.59E-05
ko00130	Ubiquinone biosynthesis	8.23E-06	4.79E-05
ko05215	Prostate cancer	1.05E-05	5.88E-05
ko00480	Glutathione metabolism	1.17E-05	6.33E-05
ko00230	Purine metabolism	1.27E-05	6.70E-05
ko00450	Selenoamino acid metabolism	1.76E-05	8.97E-05
ko05200	Pathways in cancer	2.97E-05	0.000146709
ko00030	Pentose phosphate pathway	3.25E-05	0.000155638
ko05218	Melanoma	4.96E-05	0.000231137
ko04010	MAPK signaling pathway	7.54E-05	0.000341198
ko04113	Meiosis - yeast	0.000113157	0.000485385
ko05214	Glioma	0.00011108	0.000485385
ko04145	Phagosome	0.000117526	0.000491197
ko00250	Alanine metabolism	0.00018127	0.000738676

ko00980	Metabolism of cytochrome P450	0.000218672	0.000858501
ko00910	Nitrogen metabolism	0.000221209	0.000858501
ko00680	Methane metabolism	0.00029613	0.001097026
ko04370	VEGF signaling pathway	0.00029613	0.001097026
ko03040	Spliceosome	0.000651454	0.002359713
ko03320	PPAR signaling pathway	0.000761622	0.002641369
ko05210	Colorectal cancer	0.000761622	0.002641369
ko04150	mTOR signaling pathway	0.00092891	0.003090046
ko04140	Regulation of autophagy	0.000921709	0.003090046
ko00941	Flavonoid biosynthesis	0.001412887	0.004606012
ko04530	Tight junction	0.001544922	0.004937692
ko00120	Primary bile acid biosynthesis	0.001661318	0.005207593
ko05110	Vibrio cholerae infection	0.001732985	0.005231049
ko04360	Axon guidance	0.001732985	0.005231049
ko05010	Alzheimer's disease	0.00198534	0.005883826
ko00052	Galactose metabolism	0.002735148	0.007821565
ko04910	Insulin signaling pathway	0.002735148	0.007821565
ko00330	Arginine and proline metabolism	0.002864544	0.008050357
ko04966	Collecting duct acid secretion	0.005029968	0.013896353
ko00860	Porphyrin and chlorophyll metabolism	0.005364527	0.014334721
ko04742	Taste transduction	0.005364527	0.014334721
ko00290	Valine, leucine and isoleucine biosynthesis	0.005663658	0.014889939
ko04062	Chemokine signaling pathway	0.005969991	0.015050519
ko04142	Lysosome	0.006014721	0.015050519
ko00190	Oxidative phosphorylation	0.006094075	0.015050519
ko04540	Gap junction	0.005927983	0.015050519
ko05012	Parkinson's disease	0.006449409	0.015690353
ko02030	Bacterial chemotaxis	0.007920339	0.018985519
ko04260	Cardiac muscle contraction	0.008319353	0.019652964
ko03060	Protein export	0.008887411	0.020694972
ko04916	Melanogenesis	0.010180124	0.02304667
ko04270	Vascular smooth muscle contraction	0.010180124	0.02304667
ko00565	Ether lipid metabolism	0.013443315	0.030017264
ko00591	Linoleic acid metabolism	0.014325014	0.031553748
ko04662	B cell receptor signaling pathway	0.014662252	0.03186596
ko04146	Peroxisome	0.015878937	0.034056142
ko00950	Isoquinoline alkaloid biosynthesis	0.016576042	0.035089544
ko04722	Neurotrophin signaling pathway	0.017303955	0.03616083
ko04340	Hedgehog signaling pathway	0.01813022	0.037407922
ko05014	Amyotrophic lateral sclerosis (ALS)	0.023092855	0.047051693
ko00643	Styrene degradation	0.023722522	0.047737914
ko00350	Tyrosine metabolism	0.026926258	0.053524148
ko00380	Tryptophan metabolism	0.031135271	0.059706461

ko00040	Pentose and glucuronate interconversions	0.030804026	0.059706461
ko04912	GnRH signaling pathway	0.030804026	0.059706461
ko00020	Citrate cycle (TCA cycle)	0.031524214	0.059749382
ko04720	Long-term potentiation	0.032725848	0.061313944
ko00521	Streptomycin biosynthesis	0.036602469	0.067575585
ko05142	Chagas disease	0.036897099	0.067575585
ko00361	Hexachlorocyclohexane degradation	0.042381293	0.07675723
ko00940	Phenylpropanoid biosynthesis	0.046882375	0.082170185
ko04962	Vasopressin-regulated water reabsorption	0.046882375	0.082170185
ko04810	Regulation of actin cytoskeleton	0.046266173	0.082170185
ko04650	Natural killer cell mediated cytotoxicity	0.049739325	0.086250106
ko04310	Wnt signaling pathway	0.051120633	0.087712244
ko00600	Sphingolipid metabolism	0.054178607	0.091990761
ko04660	T cell receptor signaling pathway	0.057053153	0.095872824
ko00524	Butirosin and neomycin biosynthesis	0.060459448	0.099544343
ko04210	Apoptosis	0.060459448	0.099544343
ko05222	Small cell lung cancer	0.069492904	0.113273433
ko05212	Pancreatic cancer	0.073709334	0.116646809
ko04520	Adherens junction	0.073093937	0.116646809
ko00401	Novobiocin biosynthesis	0.073709334	0.116646809
ko00053	Ascorbate and aldarate metabolism	0.077385474	0.121286849
ko00564	Glycerophospholipid metabolism	0.078273744	0.12151067
ko00400	Phenylalanine biosynthesis	0.08064965	0.122858813
ko00062	Fatty acid elongation in mitochondria	0.08064965	0.122858813
ko04960	Aldosterone-regulated sodium reabsorption	0.087781983	0.13248577
ko00364	Fluorobenzoate degradation	0.091567135	0.13693067
ko05211	Renal cell carcinoma	0.102571154	0.150622505
ko05131	Shigellosis	0.102571154	0.150622505
ko04620	Toll-like receptor signaling pathway	0.110203479	0.157571641
ko04664	Fc epsilon RI signaling pathway	0.110203479	0.157571641
ko04012	ErbB signaling pathway	0.110203479	0.157571641
ko00626	Naphthalene and anthracene degradation	0.113117787	0.160332167
ko00901	Indole alkaloid biosynthesis	0.134157276	0.186902872
ko00471	D-Glutamine and D-glutamate metabolism	0.133914325	0.186902872
ko00410	beta-Alanine metabolism	0.143650704	0.198432752
ko00550	Peptidoglycan biosynthesis	0.154697726	0.206686306
ko00592	alpha-Linolenic acid metabolism	0.154697726	0.206686306
ko04711	Circadian rhythm - fly	0.154697726	0.206686306
ko00523	Polyketide sugar unit biosynthesis	0.154697726	0.206686306
ko05016	Huntington's disease	0.15862711	0.210213162
ko00561	Glycerolipid metabolism	0.170441298	0.224047835
ko05100	Bacterial invasion of epithelial cells	0.17475097	0.227875265
ko03020	RNA polymerase	0.188217161	0.242179558

ko00240	Pyrimidine metabolism	0.188692048	0.242179558
ko03010	Ribosome	0.194328564	0.247465281
ko00640	Propanoate metabolism	0.204006179	0.25777525
ko00520	nucleotide sugar metabolism	0.210074104	0.263400607
ko01040	Biosynthesis of unsaturated fatty acids	0.213441787	0.265580239
ko00340	Histidine metabolism	0.218818158	0.270207271
ko04320	Dorso-ventral axis formation	0.232101653	0.280241256
ko04744	Phototransduction	0.232101653	0.280241256
ko04013	MAPK signaling pathway - fly	0.232101653	0.280241256
ko04745	Phototransduction - fly	0.250318914	0.291442735
ko05219	Bladder cancer	0.250318914	0.291442735
ko05220	Chronic myeloid leukemia	0.250318914	0.291442735
ko05221	Acute myeloid leukemia	0.250318914	0.291442735
ko04740	Olfactory transduction	0.250318914	0.291442735
ko00071	Fatty acid metabolism	0.25978419	0.300317893
ko04930	Type II diabetes mellitus	0.268104065	0.301385949
ko05020	Prion diseases	0.268104065	0.301385949
ko05120	Epithelial cell signaling	0.268104065	0.301385949
ko05216	Thyroid cancer	0.268104065	0.301385949
ko00632	Benzoate degradation via CoA ligation	0.271695102	0.303330834
ko04670	Leukocyte transendothelial migration	0.302418789	0.333069342
ko05140	Leishmaniasis	0.302418789	0.333069342
ko00900	Terpenoid backbone biosynthesis	0.335124927	0.366613176
ko03430	Mismatch repair	0.341835718	0.37146148
ko04730	Long-term depression	0.434278763	0.468790983
ko00631	1,2-Dichloroethane degradation	0.477045405	0.504924682
ko00903	Limonene and pinene degradation	0.477045405	0.504924682
ko00072	Synthesis and degradation of ketone bodies	0.477045405	0.504924682
ko04626	Plant-pathogen interaction	0.501565288	0.527452529
ko04914	Progesterone-mediated oocyte maturation	0.524935681	0.544996917
ko03022	Basal transcription factors	0.524935681	0.544996917
ko04621	NOD-like receptor signaling pathway	0.547210462	0.564527249
ko00310	Lysine degradation	0.554984024	0.568945887
ko00641	3-Chloroacrylic acid degradation	0.607962768	0.61936207
ko00650	Butanoate metabolism	0.65625458	0.664406811
ko03410	Base excision repair	0.660564879	0.66464244
ko04350	TGF-beta signaling pathway	0.799866913	0.799866913

Table S3 The information of 2274 DEGs in ‘S vs F’ comparison were assigned to 174 KEGG pathways.

ko_id	ko_name	pvalue	FDR
ko00940	Phenylpropanoid biosynthesis	0	0
ko04110	Cell cycle	0	0
ko00643	Styrene degradation	0	0
ko00620	Pyruvate metabolism	0	0
ko00120	Primary bile acid biosynthesis	0	0
ko00364	Fluorobenzoate degradation	0	0
ko02030	Bacterial chemotaxis	0	0
ko00500	Starch and sucrose metabolism	9.72E-49	2.11E-47
ko00460	Cyanoamino acid metabolism	7.40E-42	1.43E-40
ko00051	Fructose and mannose metabolism	1.94E-26	3.37E-25
ko00270	Cysteine and methionine metabolism	4.81E-25	7.61E-24
ko00260	Glycine, serine and threonine metabolism	1.48E-23	2.14E-22
ko03030	DNA replication	5.72E-20	7.66E-19
ko00010	Glycolysis / Gluconeogenesis	2.22E-16	2.41E-15
ko00330	Arginine and proline metabolism	2.22E-16	2.41E-15
ko00280	Valine, leucine and isoleucine degradation	2.22E-16	2.41E-15
ko00562	Inositol phosphate metabolism	3.33E-16	3.22E-15
ko00250	Alanine, aspartate and glutamate metabolism	3.33E-16	3.22E-15
ko04111	Cell cycle - yeast	1.33E-15	1.22E-14
ko00630	Glyoxylate and dicarboxylate metabolism	2.44E-15	2.12E-14
ko00710	Carbon fixation in photosynthetic organisms	6.44E-15	5.17E-14
ko00910	Nitrogen metabolism	6.54E-15	5.17E-14
ko00905	Brassinosteroid biosynthesis	6.78E-14	5.13E-13
ko03420	Nucleotide excision repair	1.62E-12	1.17E-11
ko04115	p53 signaling pathway	2.99E-12	2.08E-11
ko00360	Phenylalanine metabolism	3.63E-12	2.43E-11
ko04070	Phosphatidylinositol signaling system	6.42E-12	4.14E-11
ko00230	Purine metabolism	1.25E-11	7.75E-11
ko00290	Valine, leucine and isoleucine biosynthesis	3.38E-11	2.03E-10
ko00670	One carbon pool by folate	6.77E-11	3.93E-10
ko04114	Oocyte meiosis	8.96E-11	5.03E-10
ko04510	Focal adhesion	1.98E-10	1.07E-09
ko00640	Propanoate metabolism	6.45E-10	3.40E-09
ko05213	Endometrial cancer	1.04E-09	5.31E-09
ko05215	Prostate cancer	2.95E-09	1.47E-08
ko00380	Tryptophan metabolism	3.96E-09	1.91E-08
ko04666	Fc gamma R-mediated phagocytosis	5.60E-09	2.63E-08
ko00030	Pentose phosphate pathway	6.29E-09	2.88E-08
ko04020	Calcium signaling pathway	9.51E-09	4.24E-08
ko00680	Methane metabolism	1.53E-08	6.67E-08

ko00941	Flavonoid biosynthesis	5.30E-08	2.25E-07
ko05200	Pathways in cancer	5.88E-08	2.43E-07
ko05218	Melanoma	1.08E-07	4.39E-07
ko04145	Phagosome	1.28E-07	5.06E-07
ko00480	Glutathione metabolism	1.46E-07	5.63E-07
ko04142	Lysosome	2.11E-07	7.98E-07
ko05010	Alzheimer's disease	2.30E-07	8.50E-07
ko04360	Axon guidance	4.07E-07	1.48E-06
ko04910	Insulin signaling pathway	4.21E-07	1.50E-06
ko00521	Streptomycin biosynthesis	4.64E-07	1.62E-06
ko00130	Ubiquinone biosynthesis	5.06E-07	1.73E-06
ko05214	Glioma	6.15E-07	2.06E-06
ko05012	Parkinson's disease	7.73E-07	2.54E-06
ko03320	PPAR signaling pathway	1.02E-06	3.29E-06
ko00410	beta-Alanine metabolism	1.21E-06	3.83E-06
ko00071	Fatty acid metabolism	1.79E-06	5.56E-06
ko00632	Benzoate degradation via CoA ligation	2.22E-06	6.78E-06
ko00190	Oxidative phosphorylation	2.51E-06	7.52E-06
ko00450	Selenoamino acid metabolism	2.88E-06	8.48E-06
ko00052	Galactose metabolism	3.81E-06	1.11E-05
ko05210	Colorectal cancer	4.55E-06	1.28E-05
ko04144	Endocytosis	4.50E-06	1.28E-05
ko00020	Citrate cycle (TCA cycle)	6.79E-06	1.87E-05
ko00524	Butirosin and neomycin biosynthesis	9.88E-06	2.69E-05
ko00310	Lysine degradation	1.09E-05	2.91E-05
ko00053	Ascorbate and aldarate metabolism	1.97E-05	5.21E-05
ko05110	Vibrio cholerae infection	2.01E-05	5.22E-05
ko00240	Pyrimidine metabolism	2.59E-05	6.62E-05
ko00650	Butanoate metabolism	2.77E-05	6.99E-05
ko04010	MAPK signaling pathway	3.62E-05	8.99E-05
ko04370	VEGF signaling pathway	4.62E-05	0.000113228
ko03060	Protein export	7.84E-05	0.000189472
ko04966	Collecting duct acid secretion	8.13E-05	0.000193851
ko04113	Meiosis - yeast	9.39E-05	0.000220877
ko04150	mTOR signaling pathway	0.000109899	0.000248344
ko00520	Amino sugar metabolism	0.000107147	0.000248344
ko00040	Pentose and glucuronate interconversions	0.000109534	0.000248344
ko04062	Chemokine signaling pathway	0.000137814	0.000307431
ko04120	Ubiquitin mediated proteolysis	0.000184556	0.00040649
ko04530	Tight junction	0.000277509	0.000603582
ko00631	1,2-Dichloroethane degradation	0.000320197	0.000679442
ko05014	Amyotrophic lateral sclerosis (ALS)	0.000320197	0.000679442
ko04140	Regulation of autophagy	0.000379304	0.000795168

ko04146	Peroxisome	0.000446489	0.000924871
ko04742	Taste transduction	0.000486779	0.000984878
ko00523	Polyketide sugar unit biosynthesis	0.000486779	0.000984878
ko00340	Histidine metabolism	0.000567462	0.001134925
ko04916	Melanogenesis	0.000637492	0.001246333
ko04260	Cardiac muscle contraction	0.000637492	0.001246333
ko04270	Vascular smooth muscle contraction	0.000791074	0.001529409
ko04722	Neurotrophin signaling pathway	0.000801379	0.001532306
ko00980	Metabolism of cytochrome P450	0.000903527	0.001708844
ko04810	Regulation of actin cytoskeleton	0.000973803	0.001821954
ko00860	Porphyrin and chlorophyll metabolism	0.001783375	0.003301141
ko04540	Gap junction	0.001828432	0.003348918
ko03040	Spliceosome	0.002099432	0.003765992
ko04662	B cell receptor signaling pathway	0.002080949	0.003765992
ko00641	3-Chloroacrylic acid degradation	0.002610089	0.00463424
ko00561	Glycerolipid metabolism	0.002658179	0.004671952
ko04340	Hedgehog signaling pathway	0.003376527	0.005875157
ko05131	Shigellosis	0.005608504	0.009662175
ko00361	gamma-Hexachlorocyclohexane degradation	0.007911048	0.013301421
ko00900	Terpenoid backbone biosynthesis	0.007911048	0.013301421
ko00950	Isoquinoline alkaloid biosynthesis	0.008026719	0.013301421
ko05142	Chagas disease	0.008026719	0.013301421
ko04912	GnRH signaling pathway	0.008652424	0.014203035
ko00471	D-Glutamine and D-glutamate metabolism	0.008845255	0.014383873
ko04960	Aldosterone-regulated sodium reabsorption	0.010410676	0.016525625
ko00350	Tyrosine metabolism	0.010344277	0.016525625
ko04310	Wnt signaling pathway	0.010447234	0.016525625
ko05212	Pancreatic cancer	0.010884423	0.016896387
ko00400	Phenylalanine biosynthesis	0.010972941	0.016896387
ko00401	Novobiocin biosynthesis	0.010972941	0.016896387
ko04711	Circadian rhythm - fly	0.013045284	0.019911222
ko04620	Toll-like receptor signaling pathway	0.013234742	0.020024741
ko04720	Long-term potentiation	0.014238025	0.021357038
ko04660	T cell receptor signaling pathway	0.014428103	0.021457179
ko04664	Fc epsilon RI signaling pathway	0.015917892	0.023472146
ko04962	Vasopressin-regulated water reabsorption	0.01727949	0.025265809
ko05211	Renal cell carcinoma	0.018954227	0.027033078
ko05016	Huntington's disease	0.018954227	0.027033078
ko04012	ErbB signaling pathway	0.018954227	0.027033078
ko04650	Natural killer cell mediated cytotoxicity	0.019546956	0.027384831
ko00591	Linoleic acid metabolism	0.019673011	0.027384831
ko00072	Synthesis and degradation of ketone bodies	0.019573449	0.027384831
ko04520	Adherens junction	0.021726675	0.030003503

ko00281	Geraniol degradation	0.024556952	0.033644958
ko05222	Small cell lung cancer	0.032775605	0.044554337
ko00903	Limonene and pinene degradation	0.038615466	0.052085978
ko04320	Dorso-ventral axis formation	0.048614754	0.064083085
ko05100	Bacterial invasion of epithelial cells	0.048614754	0.064083085
ko04013	MAPK signaling pathway - fly	0.048614754	0.064083085
ko00550	Peptidoglycan biosynthesis	0.051826732	0.067297398
ko00592	alpha-Linolenic acid metabolism	0.051826732	0.067297398
ko03430	Mismatch repair	0.057826029	0.074531326
ko05020	Prion diseases	0.061162025	0.078251414
ko04930	Type II diabetes mellitus	0.075045252	0.094622274
ko00600	Sphingolipid metabolism	0.075045252	0.094622274
ko05219	Bladder cancer	0.090173926	0.112073308
ko05120	Epithelial cell signaling	0.090173926	0.112073308
ko00061	Fatty acid biosynthesis	0.10644841	0.126863173
ko05220	Chronic myeloid leukemia	0.10644841	0.126863173
ko05140	Leishmaniasis	0.10644841	0.126863173
ko04210	Apoptosis	0.10644841	0.126863173
ko05221	Acute myeloid leukemia	0.10644841	0.126863173
ko05216	Thyroid cancer	0.10644841	0.126863173
ko03440	Homologous recombination	0.124216051	0.146037789
ko01040	Biosynthesis of unsaturated fatty acids	0.124216051	0.146037789
ko04670	Leukocyte transendothelial migration	0.161076021	0.188102199
ko00565	Ether lipid metabolism	0.171162383	0.198548364
ko00440	Phosphonate and phosphinate metabolism	0.216089824	0.249004168
ko00564	Glycerophospholipid metabolism	0.218308335	0.249905594
ko04730	Long-term depression	0.229946372	0.261507639
ko04745	Phototransduction - fly	0.239282543	0.266892067
ko04740	Olfactory transduction	0.239282543	0.266892067
ko04744	Phototransduction	0.239282543	0.266892067
ko04621	NOD-like receptor signaling pathway	0.253995459	0.281498152
ko00626	Naphthalene and anthracene degradation	0.277194563	0.305264899
ko03410	Base excision repair	0.307499721	0.336509129
ko00603	Glycosphingolipid biosynthesis - globo series	0.328591236	0.357342969
ko00901	Indole alkaloid biosynthesis	0.385486911	0.416613183
ko04350	TGF-beta signaling pathway	0.39611122	0.425452791
ko00062	Fatty acid elongation in mitochondria	0.414825954	0.44012022
ko00604	Glycosphingolipid biosynthesis - ganglio series	0.414825954	0.44012022
ko04626	Plant-pathogen interaction	0.480452019	0.500590727
ko04914	Progesterone-mediated oocyte maturation	0.480452019	0.500590727
ko03010	Ribosome	0.477555437	0.500590727
ko03020	RNA polymerase	0.521128491	0.536546494

ko00830	Retinol metabolism	0.518280189	0.536546494
ko00511	Other glycan degradation	0.633067155	0.647962853
ko03022	Basal transcription factors	0.707463816	0.71569014
ko00531	Glycosaminoglycan degradation	0.707463816	0.71569014
ko00720	Reductive carboxylate cycle (CO2 fixation)	0.727058233	0.731260882
ko04612	Antigen processing and presentation	0.802720945	0.802720945

Table S4 The information of 3534 DEGs in 'S vs L' comparison were assigned to 177 KEGG pathways.

ko_id	ko_name	pvalue	FDR
ko00380	Tryptophan metabolism	0	0
ko04530	Tight junction	0	0
ko04666	Fc gamma R-mediated phagocytosis	0	0
ko00230	Purine metabolism	0	0
ko00130	Ubiquinone biosynthesis	0	0
ko04113	Meiosis - yeast	0	0
ko00940	Phenylpropanoid biosynthesis	0	0
ko05222	Small cell lung cancer	0	0
ko00071	Fatty acid metabolism	0	0
ko04742	Taste transduction	0	0
ko00480	Glutathione metabolism	0	0
ko04962	Vasopressin-regulated water reabsorption	0	0
ko04144	Endocytosis	0	0
ko04010	MAPK signaling pathway	0	0
ko05010	Alzheimer's disease	0	0
ko00310	Lysine degradation	0	0
ko04114	Oocyte meiosis	0	0
ko00052	Galactose metabolism	0	0
ko00643	Styrene degradation	0	0
ko00190	Oxidative phosphorylation	0	0
ko00640	Propanoate metabolism	0	0
ko05012	Parkinson's disease	0	0
ko00030	Pentose phosphate pathway	0	0
ko00941	Flavonoid biosynthesis	0	0
ko00620	Pyruvate metabolism	0	0
ko00120	Primary bile acid biosynthesis	0	0
ko00630	Glyoxylate and dicarboxylate metabolism	0	0
ko00240	Pyrimidine metabolism	0	0
ko04110	Cell cycle	7.89E-135	4.82E-134
ko00500	Starch and sucrose metabolism	1.08E-119	6.37E-119
ko00010	Glycolysis / Gluconeogenesis	6.75E-118	3.86E-117
ko00051	Fructose and mannose metabolism	2.37E-90	1.31E-89
ko00270	Cysteine and methionine metabolism	9.29E-89	4.98E-88
ko00460	Cyanoamino acid metabolism	1.52E-84	7.91E-84
ko03030	DNA replication	2.38E-79	1.21E-78
ko00280	Valine, leucine and isoleucine degradation	3.30E-66	1.62E-65
ko00330	Arginine and proline metabolism	3.02E-63	1.45E-62

ko00260	Glycine, serine and threonine metabolism	7.26E-60	3.38E-59
ko04510	Focal adhesion	3.37E-52	1.53E-51
ko05200	Pathways in cancer	7.41E-48	3.28E-47
ko00710	Carbon fixation in photosynthetic organisms	5.59E-47	2.41E-46
ko03420	Nucleotide excision repair	3.38E-43	1.42E-42
ko00562	Inositol phosphate metabolism	1.40E-42	5.78E-42
ko00910	Nitrogen metabolism	2.46E-41	9.67E-41
ko00360	Phenylalanine metabolism	2.46E-41	9.67E-41
ko05213	Endometrial cancer	5.20E-40	2.00E-39
ko05215	Prostate cancer	2.45E-38	9.24E-38
ko04360	Axon guidance	1.20E-37	4.42E-37
ko05210	Colorectal cancer	2.61E-35	9.42E-35
ko04070	Phosphatidylinositol signaling system	3.74E-35	1.32E-34
ko04115	p53 signaling pathway	4.94E-33	1.71E-32
ko04020	Calcium signaling pathway	1.54E-32	5.24E-32
ko04910	Insulin signaling pathway	8.24E-32	2.75E-31
ko05218	Melanoma	1.05E-27	3.43E-27
ko04062	Chemokine signaling pathway	1.35E-26	4.34E-26
ko05214	Glioma	1.70E-25	5.38E-25
ko00670	One carbon pool by folate	1.79E-25	5.56E-25
ko00905	Brassinosteroid biosynthesis	3.64E-23	1.11E-22
ko04916	Melanogenesis	7.32E-23	2.20E-22
ko03320	PPAR signaling pathway	1.01E-22	2.99E-22
ko04370	VEGF signaling pathway	1.76E-22	5.10E-22
ko04722	Neurotrophin signaling pathway	1.88E-22	5.28E-22
ko04662	B cell receptor signaling pathway	1.88E-22	5.28E-22
ko04260	Cardiac muscle contraction	2.62E-21	7.24E-21
ko00521	Streptomycin biosynthesis	9.09E-20	2.48E-19
ko04810	Regulation of actin cytoskeleton	1.96E-19	5.27E-19
ko00632	Benzoate degradation via CoA ligation	4.03E-19	1.06E-18
ko04270	Vascular smooth muscle contraction	4.09E-19	1.06E-18
ko00680	Methane metabolism	2.00E-18	5.12E-18
ko04660	T cell receptor signaling pathway	4.17E-18	1.05E-17
ko04142	Lysosome	1.11E-16	2.66E-16
ko00250	Alanine, aspartate and glutamate metabolism	1.11E-16	2.66E-16
ko00410	beta-Alanine metabolism	1.11E-16	2.66E-16
ko04310	Wnt signaling pathway	1.11E-16	2.66E-16
ko04150	mTOR signaling pathway	2.22E-16	5.10E-16
ko04145	Phagosome	2.22E-16	5.10E-16
ko03060	Protein export	2.22E-16	5.10E-16
ko05110	Vibrio cholerae infection	3.33E-16	7.46E-16
ko00450	Selenoamino acid metabolism	3.33E-16	7.46E-16
ko04012	ErbB signaling pathway	3.92E-16	8.68E-16

ko00020	Citrate cycle (TCA cycle)	4.44E-16	9.70E-16
ko04720	Long-term potentiation	5.55E-16	1.18E-15
ko04111	Cell cycle - yeast	5.55E-16	1.18E-15
ko00650	Butanoate metabolism	6.66E-16	1.40E-15
ko00290	Valine, leucine and isoleucine biosynthesis	7.77E-16	1.62E-15
ko00561	Glycerolipid metabolism	1.98E-14	4.07E-14
ko00520	Amino sugar and nucleotide sugar metabolism	3.09E-14	6.28E-14
ko00040	Pentose and glucuronate interconversions	3.33E-14	6.70E-14
ko00053	Ascorbate and aldarate metabolism	3.43E-14	6.82E-14
ko05014	Amyotrophic lateral sclerosis (ALS)	4.60E-14	9.05E-14
ko04912	GnRH signaling pathway	9.39E-14	1.83E-13
ko04540	Gap junction	1.23E-13	2.37E-13
ko04966	Collecting duct acid secretion	1.35E-13	2.57E-13
ko04650	Natural killer cell mediated cytotoxicity	1.86E-13	3.50E-13
ko04340	Hedgehog signaling pathway	2.02E-13	3.77E-13
ko04120	Ubiquitin mediated proteolysis	4.92E-13	9.08E-13
ko00524	Butirosin and neomycin biosynthesis	9.40E-13	1.71E-12
ko04146	Peroxisome	1.54E-12	2.79E-12
ko05142	Chagas disease	3.40E-12	6.09E-12
ko04711	Circadian rhythm - fly	7.81E-12	1.37E-11
ko00523	Polyketide sugar unit biosynthesis	7.81E-12	1.37E-11
ko05212	Pancreatic cancer	1.01E-11	1.74E-11
ko00564	Glycerophospholipid metabolism	1.01E-11	1.74E-11
ko00641	3-Chloroacrylic acid degradation	1.26E-11	2.15E-11
ko03040	Spliceosome	1.89E-11	3.18E-11
ko05131	Shigellosis	1.97E-11	3.29E-11
ko03020	RNA polymerase	2.77E-11	4.58E-11
ko04620	Toll-like receptor signaling pathway	3.68E-11	5.97E-11
ko04664	Fc epsilon RI signaling pathway	3.68E-11	5.97E-11
ko05211	Renal cell carcinoma	6.58E-11	1.06E-10
ko04520	Adherens junction	1.85E-10	2.95E-10
ko00631	1,2-Dichloroethane degradation	1.90E-10	3.01E-10
ko04960	Aldosterone-regulated sodium reabsorption	2.06E-10	3.22E-10
ko04140	Regulation of autophagy	3.82E-10	5.93E-10
ko00340	Histidine metabolism	4.00E-10	6.16E-10
ko00903	Limonene and pinene degradation	6.78E-10	1.03E-09
ko00980	Metabolism of cytochrome P450	2.16E-09	3.27E-09
ko04210	Apoptosis	5.52E-09	8.28E-09
ko00361	Hexachlorocyclohexane degradation	8.08E-09	1.20E-08
ko00281	Geraniol degradation	1.13E-08	1.67E-08
ko05016	Huntington's disease	1.61E-08	2.35E-08
ko00350	Tyrosine metabolism	2.48E-08	3.60E-08
ko04730	Long-term depression	3.27E-08	4.71E-08

ko04621	NOD-like receptor signaling pathway	7.00E-08	9.99E-08
ko00471	D-Glutamine and D-glutamate metabolism	1.03E-07	1.45E-07
ko04320	Dorso-ventral axis formation	1.25E-07	1.74E-07
ko04013	MAPK signaling pathway - fly	1.25E-07	1.74E-07
ko03430	Mismatch repair	1.88E-07	2.60E-07
ko05219	Bladder cancer	2.44E-07	3.22E-07
ko05220	Chronic myeloid leukemia	2.44E-07	3.22E-07
ko05140	Leishmaniasis	2.44E-07	3.22E-07
ko05020	Prion diseases	2.44E-07	3.22E-07
ko05221	Acute myeloid leukemia	2.44E-07	3.22E-07
ko05216	Thyroid cancer	2.44E-07	3.22E-07
ko04930	Type II diabetes mellitus	4.43E-07	5.80E-07
ko00626	Naphthalene and anthracene degradation	4.50E-07	5.86E-07
ko00550	Peptidoglycan biosynthesis	2.20E-06	2.85E-06
ko04350	TGF-beta signaling pathway	5.71E-06	7.33E-06
ko00565	Ether lipid metabolism	6.07E-06	7.72E-06
ko00950	Isoquinoline alkaloid biosynthesis	8.55E-06	1.08E-05
ko04914	Progesterone-mediated oocyte maturation	1.57E-05	1.98E-05
ko00591	Linoleic acid metabolism	2.90E-05	3.56E-05
ko00900	Terpenoid backbone biosynthesis	2.90E-05	3.56E-05
ko05100	Bacterial invasion of epithelial cells	2.90E-05	3.56E-05
ko00400	Phenylalanine biosynthesis	4.01E-05	4.83E-05
ko00401	Novobiocin biosynthesis	4.01E-05	4.83E-05
ko00072	Synthesis and degradation of ketone bodies	4.01E-05	4.83E-05
ko04670	Leukocyte transendothelial migration	5.11E-05	6.07E-05
ko05120	Epithelial cell signaling	5.11E-05	6.07E-05
ko00061	Fatty acid biosynthesis	5.56E-05	6.56E-05
ko00531	Glycosaminoglycan degradation	8.58E-05	0.000101
ko04626	Plant-pathogen interaction	0.00013	0.000152
ko00592	alpha-Linolenic acid metabolism	0.000167	0.000193
ko00860	Porphyrin and chlorophyll metabolism	0.000328	0.000377
ko00603	Glycosphingolipid biosynthesis - globo series	0.000489	0.000558
ko03410	Base excision repair	0.000623	0.000707
ko01040	Biosynthesis of unsaturated fatty acids	0.000883	0.000995
ko00604	Glycosphingolipid biosynthesis	0.001029	0.001153
ko04745	Phototransduction - fly	0.001299	0.001419
ko00830	Retinol metabolism	0.001299	0.001419
ko04740	Olfactory transduction	0.001299	0.001419
ko04744	Phototransduction	0.001299	0.001419
ko00364	Fluorobenzoate degradation	0.001979	0.002149
ko03018	RNA degradation	0.003889	0.004172
ko02030	Bacterial chemotaxis	0.003889	0.004172
ko00600	Sphingolipid metabolism	0.004091	0.004362

ko00511	Other glycan degradation	0.007203	0.007635
ko00062	Fatty acid elongation in mitochondria	0.010598	0.011166
ko03440	Homologous recombination	0.016937	0.017738
ko00624	1- and 2-Methylnaphthalene degradation	0.021404	0.022286
ko04612	Antigen processing and presentation	0.037279	0.038587
ko03022	Basal transcription factors	0.04558	0.046905
ko00440	Phosphonate and phosphinate metabolism	0.075728	0.077479
ko00901	Indole alkaloid biosynthesis	0.145721	0.148234
ko00253	Tetracycline biosynthesis	0.167854	0.168808
ko03010	Ribosome	0.167854	0.168808
ko00720	Reductive carboxylate cycle	0.359978	0.359978

Table S5 The information of unigenes associated with fructose and mannose metabolism.

gene	Definition	EC
comp204027_c0_seq1	L-iditol 2-dehydrogenase	EC:1.1.1.14
comp213877_c0_seq1	L-iditol 2-dehydrogenase	EC:1.1.1.14
comp328462_c0_seq1	L-iditol 2-dehydrogenase	EC:1.1.1.14
comp222584_c0_seq1	L-iditol 2-dehydrogenase	EC:1.1.1.14
comp154018_c0_seq3	xylose isomerase	EC:5.3.1.5
comp157288_c0_seq2	xylose isomerase	EC:5.3.1.5
comp114034_c0_seq1	fructokinase	EC:2.7.1.4
comp155514_c0_seq2	fructokinase	EC:2.7.1.4
comp155621_c1_seq3	fructokinase	EC:2.7.1.4
comp155883_c0_seq2	fructokinase	EC:2.7.1.4
comp163149_c0_seq7	fructokinase	EC:2.7.1.4
comp163873_c1_seq1	fructokinase	EC:2.7.1.4
comp167447_c3_seq2	fructokinase	EC:2.7.1.4
comp171646_c0_seq11	fructokinase	EC:2.7.1.4
comp202207_c0_seq1	fructokinase	EC:2.7.1.4
comp204332_c0_seq1	fructokinase	EC:2.7.1.4
comp223679_c0_seq1	fructokinase	EC:2.7.1.4
comp265399_c0_seq1	fructokinase	EC:2.7.1.4
comp154845_c0_seq1	hexokinase	EC:2.7.1.1
comp160938_c0_seq5	hexokinase	EC:2.7.1.1
comp169307_c1_seq6	hexokinase	EC:2.7.1.1
comp170038_c1_seq5	hexokinase	EC:2.7.1.1
comp175141_c0_seq8	hexokinase	EC:2.7.1.1
comp246751_c0_seq1	hexokinase	EC:2.7.1.1
comp9428_c0_seq1	hexokinase	EC:2.7.1.1
comp169834_c0_seq1	mannose-6-phosphate isomerase	EC:5.3.1.8
comp219205_c0_seq1	mannose-6-phosphate isomerase	EC:5.3.1.8
comp273108_c0_seq1	mannose-6-phosphate isomerase	EC:5.3.1.8
comp28252_c0_seq1	mannose-6-phosphate isomerase	EC:5.3.1.8
comp162252_c0_seq1	mannose-1-phosphate guanylyltransferase	EC:2.7.7.22
comp166070_c0_seq7	mannose-1-phosphate guanylyltransferase	EC:2.7.7.22
comp273647_c0_seq1	phosphomannomutase	EC:5.4.2.8
comp330007_c0_seq1	phosphomannomutase	EC:5.4.2.8
comp166582_c0_seq1	GDPmannose 4,6-dehydratase	EC:4.2.1.47
comp231235_c0_seq1	GDPmannose 4,6-dehydratase	EC:4.2.1.47
comp159836_c1_seq3	GDP-L-fucose synthase	EC:1.1.1.271
comp269079_c0_seq1	GDP-L-fucose synthase	EC:1.1.1.271
comp151525_c0_seq5	fructose-1,6-bisphosphatase I	EC:3.1.3.11
comp165106_c1_seq3	fructose-1,6-bisphosphatase I	EC:3.1.3.11
comp237265_c0_seq1	fructose-1,6-bisphosphatase I	EC:3.1.3.11

comp287874_c0_seq1	fructose-1,6-bisphosphatase I	EC:3.1.3.11
comp3267_c0_seq1	fructose-1,6-bisphosphatase I	EC:3.1.3.11
comp332052_c0_seq1	fructose-1,6-bisphosphatase I	EC:3.1.3.11
comp129183_c0_seq1	6-phosphofructokinase	EC:2.7.1.11
comp138368_c0_seq2	6-phosphofructokinase	EC:2.7.1.11
comp157302_c0_seq2	6-phosphofructokinase	EC:2.7.1.11
comp159154_c0_seq3	6-phosphofructokinase	EC:2.7.1.11
comp159971_c0_seq1	6-phosphofructokinase	EC:2.7.1.11
comp174193_c3_seq1	6-phosphofructokinase	EC:2.7.1.11
comp287378_c0_seq1	6-phosphofructokinase	EC:2.7.1.11
comp324516_c0_seq1	6-phosphofructokinase	EC:2.7.1.11
comp151573_c0_seq1	pyrophosphate--fructose-6-phosphate 1-phosphotransferase	EC:2.7.1.90
comp161428_c0_seq5	pyrophosphate--fructose-6-phosphate 1-phosphotransferase	EC:2.7.1.90
comp170994_c0_seq8	pyrophosphate--fructose-6-phosphate 1-phosphotransferase	EC:2.7.1.90

Table S6 The unigene Ids of all full-length glycosyltransferase encoding genes.

Glucosyltransferase	Fucosyltransferase	Mannosyltransferase	Xylosyltransferase
comp150698	comp160879	comp159560	comp162400
comp153299	comp165810	comp165057	comp166190
comp157536	comp168494	comp168200	comp166485
comp158106	comp168774	comp168519	comp167289
comp158412	comp172219	comp170333	comp169210
comp158452	comp173274	comp171119	comp170549
comp160059	comp173657	comp172120	comp172445
comp160071	comp153886	comp173497	comp174029
comp160541		comp152958	comp174175
comp160854		comp156461	
comp160785			
comp162816			
comp163203			
comp163233			
comp163990			
comp165098			
comp165817			
comp166485			
comp167495			
comp167707			
comp167814			
comp167850			
comp168652			
comp169097			
comp169801			
comp170595			
comp172163			
comp172183			
comp172314			
comp172330			
comp172711			
comp173002			
comp173876			
comp173979			
comp174398			

Table S7 The information of unigenes associated with upstream elements of alkaloid biosynthetic pathway.

Shikimate pathways		
**3-deoxy-D-arabinoheptulosonate-7-phosphate synthase**	DHS	2.5.1.54
comp146288_c0_seq1		
comp80950_c0_seq1		
comp319520_c0_seq1		
comp314257_c0_seq1		
comp249743_c0_seq1		
comp238004_c0_seq1		
comp216125_c0_seq1		
comp173830_c3_seq9		
comp163269_c0_seq2		
comp154773_c0_seq1		
**3-dehydroquinate synthase**	DHQS	4.2.3.4
comp170632_c0_seq2		
**3-dehydroquiiiate acid dehydratase**	DHD	4.2.1.10
comp169261_c0_seq6		
**Shikimate dehydrogenase**	SKDH	1.1.1.25
comp256348_c0_seq1		
comp324931_c0_seq1		
**5-enolpyruvylshikimate-3-phosphate synthase**	SHKG	2.5.1.19
comp164334_c0_seq3		
comp259033_c0_seq1		
**Famesyl diphosphase synthase**	FPS	2.5.1.10
comp153312_c0_seq1		
MVA and MEP pathways		
**Acetyl-CoA acetyltransferase**	AACT	2.3.1.9
comp92782_c0_seq1		
comp84203_c1_seq1		
comp82004_c0_seq1		
comp5417_c0_seq1		
comp335652_c0_seq1		
comp326122_c0_seq1		
comp318901_c0_seq1		
comp271638_c0_seq1		
comp260478_c0_seq1		
comp242763_c0_seq1		
comp207879_c0_seq1		
comp167427_c0_seq4		
**HMG-CoA synthase**	HMGS	2.3.3.10
comp173724_c0_seq10		

comp297236_c0_seq1		
comp290170_c0_seq1		
**HMG-CoA reductase**	HMGR	1.1.1.34
comp245507_c0_seq1		
comp306381_c0_seq1		
comp271005_c0_seq1		
**Mevalonate kinase**	MVK	2.7.1.36
comp172280_c1_seq16		
**Phosphomevelonate kinase**	PMK	2.7.4.2
comp174050_c0_seq10		
comp174362_c1_seq6		
**Mevalonate diphosphase decarboxylase**	MVD	4.1.1.33
comp158115_c0_seq6		
comp162193_c0_seq2		
**IPP isomerase**	IPI	5.3.3.2
comp167651_c0_seq1		
comp146327_c0_seq6		
comp267159_c0_seq1		
**1-deoxyxylulose-5-phosphate synthetase**	DXS	2.2.1.7
comp214680_c0_seq1		
comp160370_c0_seq4		
comp123721_c0_seq1		
comp339150_c0_seq1		
**1-deoxy-D-xylulose-5-phosphate reductoisomerase**	DXR	1.1.1.267
comp170408_c2_seq1		
**4-diphosphocytidyl-2C-methyl-D-erythritol synthase**	CMS	2.7.7.60
comp160965_c0_seq12		
comp161183_c0_seq8		
**4-diphosphocytidyl-2C-methyl-D-erythritol kinase**	CMK	2.7.1.148
comp170701_c1_seq4		
**2-C-methyl-D-erythritol 2,4-cyclodiphosphate synthase**	MCS	4.6.1.12
comp159185_c1_seq2		
**1-hydroxy-2-methyl-2-(E)-butenyl-4-diphosphate syntfiase**	HDS	1.17.7.1
comp169618_c0_seq2		
Strictosidine biosynthesis pathway		
**Geraniol 10-hydroxylase**	G10H	1.14.14.1
comp194969_c0_seq1		
**β-subunit of tryptophan synthase**	TSB	4.2.1.20
comp250606_c0_seq1		
comp171747_c0_seq4		
comp169961_c0_seq6		

**Tryptophan decarboxylase**	TDC	4.1.1.28
comp170279_c0_seq5		
comp156186_c1_seq5		
comp155238_c0_seq1		
comp152458_c0_seq4		
comp170378_c0_seq1		
**Strictosidine synthase**	STR	4.3.3.2
comp173139_c1_seq10		
comp168757_c2_seq7		
comp166902_c1_seq1		
comp164596_c0_seq1		
comp163886_c1_seq1		
comp147772_c0_seq2		
comp144024_c0_seq2		
comp138101_c0_seq1		
comp87746_c0_seq1		

Table S8 The information of 29 putative transcripts associated with five independent transaminases.

Alanine, aspartate and glutamate metabolism	Length
comp98065_c0_seq1	208
comp326990_c0_seq1	246
comp275062_c0_seq1	250
comp253726_c0_seq1	300
comp216434_c0_seq1	566
comp214946_c0_seq1	332
comp174359_c0_seq4	4379
comp164354_c0_seq4	1829
comp160426_c1_seq1	1816
comp128519_c0_seq1	645
Glycine, serine and threonine metabolism	
comp340240_c0_seq1	213
comp320979_c0_seq1	283
comp308648_c0_seq1	324
comp236129_c0_seq1	257
comp166169_c0_seq2	1771
Porphyrin and chlorophyll metabolism	
comp317591_c0_seq1	227
comp293473_c0_seq1	230
comp22345_c0_seq1	275
comp157426_c1_seq3	2126
Valine, leucine and isoleucine degradation	
comp165325_c0_seq1	1608
comp164355_c0_seq4	1775
comp161621_c0_seq7	1538
comp160233_c0_seq1	1098
comp159370_c0_seq1	1377
comp158463_c0_seq14	2036
comp152773_c0_seq4	869
comp144629_c0_seq1	1246
comp142753_c0_seq5	470
Tyrosine metabolism	
comp159333_c0_seq2	1017

Table S9 The information of 236 sequences identified as putative P450 superfamily member.

gene	Length	Pfam_des
comp160834_c0_seq1	1941	F6KWJ2_9ROSI/49-507 F6KWJ2.1 PF00067.17;p450;
comp170242_c0_seq1	1848	Q76M94_ASPOF/29-489 Q76M94.1 PF00067.17;p450;
comp165374_c0_seq2	1752	A5BES1_VITVI/64-511 A5BES1.1 PF00067.17;p450;
comp165188_c0_seq1	1399	B8AJT8_ORYSI/37-492 B8AJT8.1 PF00067.17;p450;
comp162842_c0_seq11	2093	D6PAY1_9ASPA/37-532 D6PAY1.1 PF00067.17;p450;
comp166525_c1_seq1	1294	B9GNS3_POPTR/30-305 B9GNS3.1 PF00067.17;p450;
comp168548_c1_seq7	2418	D7SHG6_VITVI/2-450 D7SHG6.1 PF00067.17;p450;
comp160765_c0_seq8	1583	D0EYH3_TRIPR/29-492 D0EYH3.1 PF00067.17;p450;
comp168548_c1_seq6	1631	I1MPZ6_SOYBN/45-507 I1MPZ6.1 PF00067.17;p450;
comp161287_c0_seq14	1801	A9ZT56_COPJA/77-514 A9ZT56.1 PF00067.17;p450;
comp165084_c0_seq2	2591	F2DEU1_HORVD/52-481 F2DEU1.1 PF00067.17;p450;
comp166371_c1_seq7	1948	F6GTQ8_VITVI/46-506 F6GTQ8.1 PF00067.17;p450;
comp158443_c1_seq2	1948	Q8VZY3_MUSAC/36-480 Q8VZY3.1 PF00067.17;p450;
comp166998_c1_seq10	1853	A9ZT56_COPJA/77-514 A9ZT56.1 PF00067.17;p450;
comp163642_c0_seq1	1632	F2DZW0_HORVD/47-520 F2DZW0.1 PF00067.17;p450;
comp167363_c1_seq1	1536	C71A9_SOYBN/34-492 O81970.1 PF00067.17;p450;
comp159214_c0_seq1	3186	B6SSW8_MAIZE/85-513 B6SSW8.1 PF00067.17;p450;
comp160944_c0_seq1	1619	A5BLL1_VITVI/46-513 A5BLL1.1 PF00067.17;p450;
comp163055_c0_seq4	1526	Q6QHK2_ALLCE/34-499 Q6QHK2.1 PF00067.17;p450;
comp164184_c0_seq14	2662	B9RY37_RICCO/39-464 B9RY37.1 PF00067.17;p450;
comp171068_c1_seq10	1947	C5YE58_SORBI/36-495 C5YE58.1

		PF00067.17;p450;
comp162422_c0_seq1	1905	I1H179_BRADI/52-516 I1H179.1 PF00067.17;p450;
comp158671_c0_seq2	1859	C5YXQ6_SORBI/86-507 C5YXQ6.1 PF00067.17;p450;
comp159778_c0_seq4	1630	Q76M94_ASPOF/29-489 Q76M94.1 PF00067.17;p450;
comp161287_c0_seq3	1588	C5XEE3_SORBI/101-531 C5XEE3.1 PF00067.17;p450;
comp142101_c0_seq1	1636	C7D55_HYOMU/33-492 A6YIH8.1 PF00067.17;p450;
comp164916_c1_seq3	1425	A9ZT58_COPJA/33-492 A9ZT58.1 PF00067.17;p450;
comp167330_c1_seq8	1484	B9HWV6_POPTR/79-508 B9HWV6.1 PF00067.17;p450;
comp156549_c0_seq1	2170	-
comp170400_c1_seq6	1661	F6GZP7_VITVI/56-515 F6GZP7.1 PF00067.17;p450;
comp173036_c0_seq11	2487	B9GX98_POPTR/57-507 B9GX98.1 PF00067.17;p450;
comp173068_c1_seq30	2717	C5XW56_SORBI/87-568 C5XW56.1 PF00067.17;p450;
comp166422_c0_seq1	2046	F6HL72_VITVI/35-503 F6HL72.1 PF00067.17;p450;
comp163250_c0_seq27	1905	B9IC23_POPTR/39-499 B9IC23.1 PF00067.17;p450;
comp163250_c0_seq16	2037	B9IC23_POPTR/39-499 B9IC23.1 PF00067.17;p450;
comp165084_c0_seq6	1859	Q8H848_ORYSJ/51-480 Q8H848.1 PF00067.17;p450;
comp173697_c0_seq16	2017	B2LUN8_9ASPA/33-489 B2LUN8.2 PF00067.17;p450;
comp174313_c0_seq1	2111	I0DHH0_CYMEN/316-491 I0DHH0.1 PF00067.17;p450;
comp160263_c1_seq1	2679	E3VWA8_LINUS/21-472 E3VWA8.2 PF00067.17;p450;
comp165188_c0_seq3	1679	I1M9J8_SOYBN/39-500 I1M9J8.1 PF00067.17;p450;
comp158966_c0_seq2	3036	C5X7U3_SORBI/298-523 C5X7U3.1 PF00667.15;FAD_binding_1;
comp166525_c0_seq4	1808	B9R738_RICCO/30-493 B9R738.1 PF00067.17;p450;
comp163479_c1_seq2	2126	A9ZMH5_IRIHO/35-484 A9ZMH5.1

		PF00067.17;p450;
comp168447_c0_seq4	1533	C5YE58_SORBI/36-495 C5YE58.1 PF00067.17;p450;
comp159778_c0_seq1	1804	Q76M94_ASPOF/29-489 Q76M94.1 PF00067.17;p450;
comp161287_c0_seq16	1770	C5XEE4_SORBI/84-518 C5XEE4.1 PF00067.17;p450;
comp148672_c0_seq1	2362	F6HLR0_VITVI/41-482 F6HLR0.1 PF00067.17;p450;
comp157867_c0_seq6	2467	C7D55_HYOMU/33-492 A6YIH8.1 PF00067.17;p450;
comp172097_c1_seq10	1564	E4MWV2_THEHA/36-471 E4MWV2.1 PF00067.17;p450;
comp150713_c0_seq1	1558	F1CJJ0_9ERIC/36-505 F1CJJ0.1 PF00067.17;p450;
comp141341_c0_seq1	1688	B9ID94_POPTR/87-520 B9ID94.1 PF00067.17;p450;
comp165084_c0_seq1	1927	Q8H848_ORYSJ/51-480 Q8H848.1 PF00067.17;p450;
comp165188_c0_seq6	1615	G7KAS9_MEDTR/36-498 G7KAS9.1 PF00067.17;p450;
comp151089_c0_seq2	1639	F6HDD1_VITVI/2-365 F6HDD1.1 PF00067.17;p450;
comp154368_c0_seq13	1908	B9MVD1_POPTR/40-497 B9MVD1.1 PF00067.17;p450;
comp173068_c1_seq9	2534	D7TUE2_VITVI/86-564 D7TUE2.1 PF00067.17;p450;
comp171068_c1_seq8	2024	C5YE58_SORBI/36-495 C5YE58.1 PF00067.17;p450;
comp150946_c0_seq4	2154	F6HKE4_VITVI/86-531 F6HKE4.1 PF00067.17;p450;
comp164129_c0_seq3	1716	C5Z6M7_SORBI/39-508 C5Z6M7.1 PF00067.17;p450;
comp167851_c0_seq1	2041	G7L4Q9_MEDTR/38-502 G7L4Q9.1 PF00067.17;p450;
comp163968_c1_seq6	1809	F6HB31_VITVI/1-243 F6HB31.1 PF00067.17;p450;
comp158834_c0_seq1	1903	A5AK80_VITVI/17-481 A5AK80.1 PF00067.17;p450;
comp158966_c0_seq1	2391	C5X7U3_SORBI/298-523 C5X7U3.1 PF00667.15;FAD_binding_1;
comp171295_c0_seq4	1954	I1KYH8_SOYBN/4-463 I1KYH8.1 PF00067.17;p450;

comp161892_c0_seq2	1913	I1HCI5_BRADI/79-516 I1HCI5.1 PF00067.17;p450;
comp144304_c0_seq1	1639	B9RY37_RICCO/39-464 B9RY37.1 PF00067.17;p450;
comp164754_c1_seq1	1976	Q5CCK2_ORYSJ/43-492 Q5CCK2.1 PF00067.17;p450;
comp158583_c0_seq1	1806	I1QTC9_ORYGL/42-513 I1QTC9.1 PF00067.17;p450;
comp174543_c2_seq1	1692	F6I533_VITVI/29-488 F6I533.1 PF00067.17;p450;
comp162618_c0_seq1	1934	B9R737_RICCO/27-484 B9R737.1 PF00067.17;p450;
comp174016_c0_seq6	2051	A9ZT56_COPJA/77-514 A9ZT56.1 PF00067.17;p450;
comp163250_c0_seq24	2127	G7ID37_MEDTR/38-501 G7ID37.1 PF00067.17;p450;
comp159778_c0_seq3	2222	Q76M94_ASPOF/29-489 Q76M94.1 PF00067.17;p450;
comp170650_c0_seq6	1729	B9HWV4_POPTR/79-509 B9HWV4.1 PF00067.17;p450;
comp161287_c0_seq2	1646	A9ZT56_COPJA/77-514 A9ZT56.1 PF00067.17;p450;
comp161335_c0_seq2	1572	E0CQG3_VITVI/55-488 E0CQG3.1 PF00067.17;p450;
comp163250_c0_seq20	2643	G7ID36_MEDTR/196-374 G7ID36.1 PF00067.17;p450;
comp164184_c0_seq5	1778	B9RY37_RICCO/39-464 B9RY37.1 PF00067.17;p450;
comp163250_c0_seq17	2315	G7ID36_MEDTR/196-374 G7ID36.1 PF00067.17;p450;
comp159812_c0_seq1	1845	F5A637_9ASPA/30-488 F5A637.1 PF00067.17;p450;
comp166422_c0_seq2	2348	F6HL72_VITVI/35-503 F6HL72.1 PF00067.17;p450;
comp156764_c3_seq7	1762	I1L3R8_SOYBN/42-473 I1L3R8.1 PF00067.17;p450;
comp160765_c0_seq5	1774	D0EYH3_TRIPR/29-492 D0EYH3.1 PF00067.17;p450;
comp162972_c0_seq3	1892	Q6QHK2_ALLCE/34-499 Q6QHK2.1 PF00067.17;p450;
comp166758_c0_seq4	1908	B9GKM6_POPTR/31-463 B9GKM6.1 PF00067.17;p450;
comp165358_c2_seq1	1785	F6GZP7_VITVI/56-515 F6GZP7.1 PF00067.17;p450;

comp160765_c0_seq2	1537	C5Z0A1_SORBI/32-491 C5Z0A1.1 PF00067.17;p450;
comp165060_c0_seq4	1861	D7TZZ4_VITVI/36-463 D7TZZ4.1 PF00067.17;p450;
comp167090_c1_seq2	1675	A1XEL5_TOBAC/26-488 A1XEL5.1 PF00067.17;p450;
comp155354_c0_seq1	1921	A9ZMH5_IRIHO/35-484 A9ZMH5.1 PF00067.17;p450;
comp162319_c0_seq8	2913	B9GW74_POPTR/31-473 B9GW74.1 PF00067.17;p450;
comp159214_c0_seq6	2990	B6SSW8_MAIZE/85-513 B6SSW8.1 PF00067.17;p450;
comp164325_c1_seq5	1859	B9A9Y6_TRIHR/30-489 B9A9Y6.1 PF00067.17;p450;
comp165358_c2_seq2	1849	F6GZP7_VITVI/56-515 F6GZP7.1 PF00067.17;p450;
comp169784_c1_seq1	2170	Q43071_9ASPA/1-420 Q43071.1 PF00067.17;p450;
comp167524_c1_seq1	1604	B9RBQ8_RICCO/47-505 B9RBQ8.1 PF00067.17;p450;
comp165084_c0_seq11	2003	Q8H848_ORYSJ/51-480 Q8H848.1 PF00067.17;p450;
comp173068_c1_seq4	1619	D7TUE2_VITVI/86-564 D7TUE2.1 PF00067.17;p450;
comp162972_c0_seq5	1651	Q6QHK2_ALLCE/34-499 Q6QHK2.1 PF00067.17;p450;
comp173068_c1_seq47	2210	D7TUE2_VITVI/86-564 D7TUE2.1 PF00067.17;p450;
comp163935_c2_seq2	1623	I1L8I0_SOYBN/35-164 I1L8I0.1 PF08240.7;ADH_N;
comp163250_c0_seq23	1611	G7ID37_MEDTR/38-501 G7ID37.1 PF00067.17;p450;
comp167460_c0_seq1	2138	C6ENF2_HYONI/255-473 C6ENF2.1 PF00067.17;p450;
comp163479_c1_seq1	2061	A9ZMH5_IRIHO/35-484 A9ZMH5.1 PF00067.17;p450;
comp155354_c0_seq3	2159	B9R738_RICCO/30-493 B9R738.1 PF00067.17;p450;
comp145211_c0_seq1	2127	I1HCI5_BRADI/79-516 I1HCI5.1 PF00067.17;p450;
comp157049_c0_seq2	2021	ABAH3_ORYSJ/45-473 Q0J185.1 PF00067.17;p450;
comp164184_c0_seq19	3250	B9RY37_RICCO/39-464 B9RY37.1 PF00067.17;p450;

comp173068_c1_seq53	2295	D7TUE2_VITVI/86-564 D7TUE2.1 PF00067.17;p450;
comp159778_c0_seq2	2070	Q76M94_ASPOF/29-489 Q76M94.1 PF00067.17;p450;
comp165084_c0_seq4	2071	Q8H848_ORYSJ/51-480 Q8H848.1 PF00067.17;p450;
comp156590_c0_seq1	1783	B9N3N2_POPTR/35-495 B9N3N2.1 PF00067.17;p450;
comp155976_c0_seq1	1731	I1IXF3_BRADI/30-502 I1IXF3.1 PF00067.17;p450;
comp171659_c1_seq1	2086	E3VWA8_LINUS/21-472 E3VWA8.2 PF00067.17;p450;
comp123024_c0_seq2	1821	C4JB85_MAIZE/36-501 C4JB85.1 PF00067.17;p450;
comp163055_c0_seq7	2109	Q6QHK2_ALLCE/34-499 Q6QHK2.1 PF00067.17;p450;
comp162802_c1_seq1	2017	Q8H848_ORYSJ/51-480 Q8H848.1 PF00067.17;p450;
comp154368_c0_seq15	2312	B9MVD1_POPTR/40-497 B9MVD1.1 PF00067.17;p450;
comp163250_c0_seq7	2147	B9IC23_POPTR/39-499 B9IC23.1 PF00067.17;p450;
comp174120_c2_seq2	2208	-
comp167090_c1_seq5	2433	A1XEL5_TOBAC/26-488 A1XEL5.1 PF00067.17;p450;
comp168548_c1_seq8	2487	D7SHG6_VITVI/2-450 D7SHG6.1 PF00067.17;p450;
comp161335_c0_seq1	2097	E0CQG3_VITVI/55-488 E0CQG3.1 PF00067.17;p450;
comp165798_c0_seq1	1846	I1P7E7_ORYGL/34-501 I1P7E7.1 PF00067.17;p450;
comp167802_c0_seq3	2241	D6PT25_9ROSI/35-164 D6PT25.1 PF08240.7;ADH_N;
comp174016_c0_seq7	2008	A9ZT56_COPJA/77-514 A9ZT56.1 PF00067.17;p450;
comp150946_c0_seq2	2238	F6HKE4_VITVI/86-531 F6HKE4.1 PF00067.17;p450;
comp172493_c2_seq3	1683	B9N7D9_POPTR/32-497 B9N7D9.1 PF00067.17;p450;
comp167802_c0_seq7	2145	D6PT25_9ROSI/35-164 D6PT25.1 PF08240.7;ADH_N;
comp166271_c1_seq1	2018	C5XEE4_SORBI/84-518 C5XEE4.1 PF00067.17;p450;

comp102545_c0_seq1	1593	A2Z725_ORYSI/74-543 A2Z725.1 PF00067.17;p450;
comp154769_c0_seq1	1677	B9SST9_RICCO/56-491 B9SST9.1 PF00067.17;p450;
comp163250_c0_seq15	1799	G7ID37_MEDTR/38-501 G7ID37.1 PF00067.17;p450;
comp174016_c0_seq12	1768	Q9ATU2_LOLRI/90-517 Q9ATU2.1 PF00067.17;p450;
comp161892_c0_seq30	1983	C0P5Z0_MAIZE/55-476 C0P5Z0.1 PF00067.17;p450;
comp55077_c0_seq1	1664	D7SMP3_VITVI/35-456 D7SMP3.1 PF00067.17;p450;
comp173929_c1_seq6	2593	A9ZMH5_IRIHO/35-484 A9ZMH5.1 PF00067.17;p450;
comp165060_c0_seq9	2122	D7TZZ4_VITVI/36-463 D7TZZ4.1 PF00067.17;p450;
comp165084_c0_seq19	2659	F2DEU1_HORVD/52-481 F2DEU1.1 PF00067.17;p450;
comp173068_c1_seq42	3378	D7TUE2_VITVI/86-564 D7TUE2.1 PF00067.17;p450;
comp160944_c0_seq2	1727	D7LXL2_ARALL/41-509 D7LXL2.1 PF00067.17;p450;
comp159214_c0_seq3	3088	B6SSW8_MAIZE/85-513 B6SSW8.1 PF00067.17;p450;
comp168447_c0_seq1	1610	C5YE58_SORBI/36-495 C5YE58.1 PF00067.17;p450;
comp149131_c0_seq3	1788	B9N3N2_POPTR/35-495 B9N3N2.1 PF00067.17;p450;
comp160765_c0_seq4	1560	D0EYH3_TRIPR/29-492 D0EYH3.1 PF00067.17;p450;
comp170650_c0_seq5	1827	B9HWV4_POPTR/79-509 B9HWV4.1 PF00067.17;p450;
comp148285_c0_seq1	1641	A9ZT58_COPJA/33-492 A9ZT58.1 PF00067.17;p450;
comp163250_c0_seq8	2475	B9IC23_POPTR/39-499 B9IC23.1 PF00067.17;p450;
comp137025_c0_seq1	1569	C71A1_PERAE/32-491 P24465.2 PF00067.17;p450;
comp162972_c0_seq4	2118	Q6QHK2_ALLCE/34-499 Q6QHK2.1 PF00067.17;p450;
comp174281_c1_seq2	1517	I1KYH8_SOYBN/4-463 I1KYH8.1 PF00067.17;p450;
comp165449_c1_seq1	2121	B9RG11_RICCO/37-476 B9RG11.1 PF00067.17;p450;

comp165358_c2_seq12	1751	F6GZP7_VITVI/56-515 F6GZP7.1 PF00067.17;p450;
comp173068_c1_seq38	2078	D7TUE2_VITVI/86-564 D7TUE2.1 PF00067.17;p450;
comp163460_c0_seq1	1528	Q76M94_ASPOF/29-489 Q76M94.1 PF00067.17;p450;
comp92579_c0_seq1	2007	B6QUK1_PENMQ/43-520 B6QUK1.1 PF00067.17;p450;
comp171068_c1_seq15	2196	B7E6Q0_ORYSJ/35-384 B7E6Q0.1 PF00067.17;p450;
comp162802_c0_seq2	1937	Q8H848_ORYSJ/51-480 Q8H848.1 PF00067.17;p450;
comp174016_c0_seq30	2103	A9ZT56_COPJA/77-514 A9ZT56.1 PF00067.17;p450;
comp165367_c0_seq1	2009	A5AW45_VITVI/56-518 A5AW45.1 PF00067.17;p450;
comp164916_c1_seq1	1619	A9ZT58_COPJA/33-492 A9ZT58.1 PF00067.17;p450;
comp145211_c0_seq2	2203	B9SEM0_RICCO/75-517 B9SEM0.1 PF00067.17;p450;
comp163059_c0_seq8	1923	F6HFV1_VITVI/56-511 F6HFV1.1 PF00067.17;p450;
comp161287_c0_seq10	1557	C5XEE3_SORBI/101-531 C5XEE3.1 PF00067.17;p450;
comp162842_c1_seq6	1971	G7JKD7_MEDTR/26-512 G7JKD7.1 PF00067.17;p450;
comp165401_c0_seq1	2671	C5Y1D7_SORBI/27-514 C5Y1D7.1 PF00067.17;p450;
comp162972_c0_seq10	2217	Q6QHK2_ALLCE/34-499 Q6QHK2.1 PF00067.17;p450;
comp171951_c1_seq5	2551	F6HVA0_VITVI/66-564 F6HVA0.1 PF00067.17;p450;
comp154368_c0_seq9	3534	A5AD09_VITVI/64-498 A5AD09.1 PF00067.17;p450;
comp136563_c0_seq1	1505	F6HL72_VITVI/35-503 F6HL72.1 PF00067.17;p450;
comp164147_c0_seq6	1845	A2Z8P9_ORYSI/232-518 A2Z8P9.1 PF00067.17;p450;
comp168548_c1_seq2	1909	D7SHG6_VITVI/2-450 D7SHG6.1 PF00067.17;p450;
comp163059_c0_seq1	2015	A5AW45_VITVI/56-518 A5AW45.1 PF00067.17;p450;
comp169995_c0_seq5	3458	C5X7U3_SORBI/298-523 C5X7U3.1 PF00667.15;FAD_binding_1;

comp163250_c0_seq22	2421	B9IC23_POPTR/39-499 B9IC23.1 PF00067.17;p450;
comp157507_c0_seq1	1907	I1NTF9_ORYGL/38-516 I1NTF9.1 PF00067.17;p450;
comp171068_c1_seq16	2052	C5YE58_SORBI/36-495 C5YE58.1 PF00067.17;p450;
comp164147_c0_seq4	2103	F2CXW8_HORVD/59-539 F2CXW8.1 PF00067.17;p450;
comp174435_c0_seq3	1840	B2LUN8_9ASPA/33-489 B2LUN8.2 PF00067.17;p450;
comp170173_c2_seq1	1682	A1XEL5_TOBAC/26-488 A1XEL5.1 PF00067.17;p450;
comp168075_c1_seq3	2146	F6HEA0_VITVI/32-517 F6HEA0.1 PF00067.17;p450;
comp160738_c0_seq2	2982	C7D55_HYOMU/33-492 A6YIH8.1 PF00067.17;p450;
comp154480_c0_seq1	1810	C5XH17_SORBI/43-509 C5XH17.1 PF00067.17;p450;
comp159778_c0_seq9	1699	Q76M94_ASPOF/29-489 Q76M94.1 PF00067.17;p450;
comp161024_c0_seq4	4273	F2D7V0_HORVD/33-517 F2D7V0.1 PF00067.17;p450;
comp164147_c0_seq2	1825	A2Z8P9_ORYSI/232-518 A2Z8P9.1 PF00067.17;p450;
comp168512_c2_seq3	1641	C5YLV2_SORBI/43-177 C5YLV2.1 PF08240.7;ADH_N;
comp170061_c0_seq5	2209	D7TZZ4_VITVI/36-463 D7TZZ4.1 PF00067.17;p450;
comp158984_c0_seq1	1818	A9NWW9_PICSI/76-514 A9NWW9.1 PF00067.17;p450;
comp158966_c0_seq6	2255	C5X7U3_SORBI/298-523 C5X7U3.1 PF00667.15;FAD_binding_1;
comp168237_c1_seq8	2483	D7KGM5_ARALL/107-557 D7KGM5.1 PF00067.17;p450;
comp173929_c1_seq4	1769	A9ZMH5_IRIHO/35-484 A9ZMH5.1 PF00067.17;p450;
comp163124_c0_seq1	1723	Q338Z0_ORYSJ/50-482 Q338Z0.1 PF00067.17;p450;
comp168237_c0_seq9	1512	C5XVX2_SORBI/144-596 C5XVX2.1 PF00067.17;p450;
comp171544_c1_seq16	1937	B9IC23_POPTR/39-499 B9IC23.1 PF00067.17;p450;
comp173068_c1_seq36	3232	C5XW56_SORBI/87-568 C5XW56.1 PF00067.17;p450;

comp168237_c0_seq6	2228	B8AEV1_ORYSI/121-577 B8AEV1.1 PF00067.17;p450;
comp163641_c0_seq5	1904	D7SMP3_VITVI/35-456 D7SMP3.1 PF00067.17;p450;
comp168512_c2_seq9	1700	C5YLV2_SORBI/43-177 C5YLV2.1 PF08240.7;ADH_N;
comp163124_c0_seq10	1510	I1IW83_BRADI/59-494 I1IW83.1 PF00067.17;p450;
comp169703_c0_seq2	1852	A1XEL5_TOBAC/26-488 A1XEL5.1 PF00067.17;p450;
comp171068_c1_seq11	2273	B7E6Q0_ORYSJ/35-384 B7E6Q0.1 PF00067.17;p450;
comp169995_c0_seq4	4492	C5X7U3_SORBI/298-523 C5X7U3.1 PF00667.15;FAD_binding_1;
comp166371_c1_seq11	2001	D7SHG6_VITVI/2-450 D7SHG6.1 PF00067.17;p450;
comp168237_c1_seq14	2455	B8AEV1_ORYSI/121-577 B8AEV1.1 PF00067.17;p450;
comp163124_c0_seq2	1796	I1IW83_BRADI/59-494 I1IW83.1 PF00067.17;p450;
comp171322_c0_seq1	1621	B9A9Y6_TRIHR/30-489 B9A9Y6.1 PF00067.17;p450;
comp151625_c0_seq1	1873	B5MEX7_LACSA/48-482 B5MEX7.1 PF00067.17;p450;
comp170061_c0_seq1	1634	D7TZZ4_VITVI/36-463 D7TZZ4.1 PF00067.17;p450;
comp174016_c0_seq31	1962	A9ZT56_COPJA/77-514 A9ZT56.1 PF00067.17;p450;
comp171068_c1_seq9	2050	C5YE58_SORBI/36-495 C5YE58.1 PF00067.17;p450;
comp173068_c1_seq24	2680	D7TUE2_VITVI/86-564 D7TUE2.1 PF00067.17;p450;
comp165836_c1_seq3	1579	O04163_NEPRA/34-501 O04163.2 PF00067.17;p450;
comp164200_c1_seq1	1864	F6I2B2_VITVI/61-517 F6I2B2.1 PF00067.17;p450;
comp163641_c0_seq17	1610	B8AM57_ORYSI/63-444 B8AM57.1 PF00067.17;p450;
comp173068_c1_seq27	3474	C5XW56_SORBI/87-568 C5XW56.1 PF00067.17;p450;
comp164147_c0_seq1	1975	I1I4W0_BRADI/56-540 I1I4W0.1 PF00067.17;p450;
comp173068_c1_seq3	2561	D7TUE2_VITVI/86-564 D7TUE2.1 PF00067.17;p450;

comp163745_c0_seq4	1807	A5AW45_VITVI/56-518 A5AW45.1 PF00067.17;p450;
comp163124_c1_seq10	2031	I1IW83_BRADI/59-494 I1IW83.1 PF00067.17;p450;
comp162842_c0_seq1	2164	D6PAY1_9ASPA/37-532 D6PAY1.1 PF00067.17;p450;
comp174016_c0_seq21	2193	A9ZT56_COPJA/77-514 A9ZT56.1 PF00067.17;p450;
comp173068_c1_seq44	2658	D7TUE2_VITVI/86-564 D7TUE2.1 PF00067.17;p450;
comp171544_c1_seq6	1900	A9PEH4_POPTR/39-499 A9PEH4.1 PF00067.17;p450;
comp168237_c0_seq2	1556	D7KGM5_ARALL/107-557 D7KGM5.1 PF00067.17;p450;
comp173929_c1_seq5	1689	A9ZMH5_IRIHO/35-484 A9ZMH5.1 PF00067.17;p450;
comp168237_c1_seq11	2571	B8AEV1_ORYSI/121-577 B8AEV1.1 PF00067.17;p450;
comp151625_c1_seq2	1851	B5MEX7_LACSA/48-482 B5MEX7.1 PF00067.17;p450;
comp163124_c0_seq9	1776	Q338Z0_ORYSJ/50-482 Q338Z0.1 PF00067.17;p450;
comp163124_c1_seq6	2011	I1MLS1_SOYBN/36-492 I1MLS1.1 PF00067.17;p450;
comp166371_c1_seq14	2049	F6GTQ8_VITVI/46-506 F6GTQ8.1 PF00067.17;p450;
comp168237_c1_seq1	2367	B8AEV1_ORYSI/121-577 B8AEV1.1 PF00067.17;p450;
comp174435_c0_seq1	1867	B2LUN8_9ASPA/33-489 B2LUN8.2 PF00067.17;p450;
comp162842_c1_seq3	1900	G7JKD7_MEDTR/26-512 G7JKD7.1 PF00067.17;p450;
comp163124_c0_seq16	1743	I1IW83_BRADI/59-494 I1IW83.1 PF00067.17;p450;
comp171981_c0_seq9	2843	C5XW56_SORBI/87-568 C5XW56.1 PF00067.17;p450;
